# Weighted centroid trees: a general approach to summarize phylogenies in single-labeled tumor mutation tree inference

**DOI:** 10.1093/bioinformatics/btae120

**Published:** 2024-07-10

**Authors:** Hamed Vasei, Mohammad-Hadi Foroughmand-Araabi, Amir Daneshgar

**Affiliations:** Department of Mathematical Sciences, Sharif University of Technology, Tehran 111559415, Iran; Department of Mathematical Sciences, Sharif University of Technology, Tehran 111559415, Iran; Department of Mathematical Sciences, Sharif University of Technology, Tehran 111559415, Iran

## Abstract

**Motivation:**

Tumor trees, which depict the evolutionary process of cancer, provide a backbone for discovering recurring evolutionary processes in cancer. While they are not the primary information extracted from genomic data, they are valuable for this purpose. One such extraction method involves summarizing multiple trees into a single representative tree, such as consensus trees or supertrees.

**Results:**

We define the “weighted centroid tree problem” to find the centroid tree of a set of single-labeled rooted trees through the following steps: (i) mapping the given trees into the Euclidean space, (ii) computing the weighted centroid matrix of the mapped trees, and (iii) finding the nearest mapped tree (NMTP) to the centroid matrix. We show that this setup encompasses previously studied parent–child and ancestor–descendent metrics as well as the GraPhyC and TuELiP consensus tree algorithms. Moreover, we show that, while the NMTP problem is polynomial-time solvable for the adjacency embedding, it is NP-hard for ancestry and distance mappings. We introduce integer linear programs for NMTP in different setups where we also provide a new algorithm for the case of ancestry embedding called 2-AncL2, that uses a novel weighting scheme for ancestry signals. Our experimental results show that 2-AncL2 has a superior performance compared to available consensus tree algorithms. We also illustrate our setup’s application on providing representative trees for a large real breast cancer dataset, deducing that the cluster centroid trees summarize reliable evolutionary information about the original dataset.

**Availability and implementation:**

https://github.com/vasei/WAncILP.

## 1 Introduction

Cancer is often modeled as an evolutionary stochastic process that explains inter- and intratumor heterogeneity (Marusyk and Polyak 2010). Despite the heterogeneity, there are hallmarks of cancer, characterized by repeated evolutionary events and behaviors (Hanahan and Weinberg 2011). Heterogeneity necessitates the study of individual patients using their own data, however, high-throughput sequencing data are often noisy and may contain many random artifacts (Parekh *et al.* 2016). On the other hand, the recurring patterns in cancer evolution allow one to utilize data from different patients, reduce the noise of each single data source, and infer the shared evolutionary histories more accurately. Many methods trying to incorporate multipatient data have appeared in recent years (El-Kebir *et al.* 2022).

Tumor evolutionary histories are typically depicted as trees. Various algorithms can infer these trees, resulting in multiple potential trees for a single patient (Tarabichi *et al.* 2021). Moreover, tumor trees from different patients may contain significant shared evolutionary information (Caravagna *et al.* 2018). Therefore, summarizing a collection of related tumor trees into a best-fit tree, which represents the shared structures “on average,” is beneficial for utilizing this set of evolutionarily related data. Naturally, formulating the term “on average” involves the application of distance measures.

Usually, distance measures in classical phylogeny are defined for leaf-labeled trees. Recently, several dissimilarity measures on fully labeled trees have been proposed. The MLTD (Karpov *et al.* 2019), permutation, and rearrangement distances (Bernardini *et al.* 2019, 2020) are edit distances between fully labeled trees. Bourque distance (Jahn *et al.* 2021), the similarity measure used in ConTreeDP algorithm (Fu and Schwartz 2021), and the generalized Robinson–Foulds distance for phylogenetic trees (Llabrés *et al.* 2020, 2021) are also defined through comparing bipartitions. MP3 (Ciccolella *et al.* 2021) is a similarity measure that compares mutation triplets of the two trees. L. Oesper *et al.* introduced path distance (PD), parent–child distance (PCD), ancestor–descendant distance (ADD), common ancestor set distance (CASet), and distinctly inherited set comparison distance (DISC) metrics by looking at relations present in tumor trees (DiNardo *et al.* 2020, Govek *et al.* 2022).

Consensus trees are also well-known objects in phylogenetics to summarize a set of trees (Bryant 2003). GraPhyC (Govek *et al.* 2022) is the first method to compute a consensus tree for a set of tumor trees by minimizing the total PCD. ConTreeDP (Fu and Schwartz 2021) uses bipartitions to find a consensus tree, while TuELiP (Guang *et al.* 2023) finds a consensus tree based on ancestor–descendent distance. Note that finding a consensus tree with respect to the ADD is an NP-hard (Qi and El-Kebir 2024) problem. The Multiple Consensus Tree (MCT) (Aguse *et al.* 2019) and the Multiple Choice Consensus Tree (Christensen *et al.* 2020) problems generalize the consensus tree problem to simultaneously cluster trees and infer a consensus tree for each cluster.

PhyC (Matsui *et al.* 2017) is an algorithm that creates a large reference tree by combining data from a set of patients. Then, by defining a mapping between individual trees of patients and the reference tree, a numerical vector is assigned to each individual tree. These vectors are then used to apply hierarchical clustering.

Tumor phylogeny can be represented by two main tree types: mutation trees and clonal trees. Mutation trees are single-labeled rooted trees, each vertex representing a distinct mutation, modeling the chronology of mutations that occurred during tumor growth (Kim and Simon 2014, Jahn *et al.* 2016). Clonal trees are multilabeled rooted trees where each vertex represents a tumor cell population that contains all mutations along its path to the root (El-Kebir *et al.* 2015). Combination of cells into clones may be interpreted as a way of correcting for the high error rates of single-cell sequencing (Kuipers *et al.* 2017). In this study, we model tumor evolution with mutation trees and focus our analysis on single-labeled trees.

The goal of this study is to provide a general framework that may be used to summarize a set of trees into a single tree, namely the “weighted centroid tree.” Our approach consists of three major steps: (i) mapping the given trees into the space of real matrices, (ii) finding the centroid point of these mapped images (as a mean/average matrix), and then (iii) finding the best tree-approximation of the centroid matrix as a labeled tree. For this, one naturally needs an “embedding” map for the first step and a “distance” for the averaging process. The last step is actually an optimization problem asking for the closest point in the space of labeled trees to the centroid matrix, which, hereafter, is referred to as the nearest mapped tree problem (NMTP).

Note that the three steps mentioned above describe a reduction from the centroid tree problem to the NMTP problem as a more general setup to be considered. It is interesting that this more general problem is more handy to be solved in some important cases providing better results based on our proposed setup and our experiments on synthetic and real datasets (see the performance analysis of our algorithm 2-AncL2 in Section 3.5).

Remarkably, NMTP problem also crops up in some other cases in this field of research. For instance, one may use this optimization problem to infer phylogenies directly, when a matrix containing evolutionary distances between a set of species is already provided from other sources of information or through other methods (Felsenstein 2004).

On the other hand, NMTP problem is the natural setup for approximating a set of multilabeled trees by a single-labeled representative tree or any other case when one is interested in the best (i.e. nearest) single-labeled tree to a given real matrix. This is actually used in our complementary experimental results (Supplementary Section 8).

We analyze this general setup for a variety of cases including adjacency, ancestry, and distance matrix-embedding of trees and entry-wise $L_{1}$ and $L_{2}$ norms. Within this setup, we define a family of dissimilarity measures between mutation trees as the norm of the difference between their mapped images, where this reveals that previously studied “consensus tree problems” solved by GraPhyC and TuELiP fit into our generalized framework.

In our framework, besides flexibility in embeddings and norms, we can apply weighted sums to obtain weighted centroids. In particular, we introduce the algorithm 2-AncL2, which is a modification of TuELiP, in which a more general weighting strategy is used to handle missing mutations. We have done our best to evaluate our algorithm through a comprehensive analysis in comparison to other existing algorithms, both on simulated and real datasets. For clarity and brevity, all the proofs for the theorems are provided in the Supplementary Material.

## 2 Materials and methods

### 2.1 Preliminaries and notations

**Figure 1.**
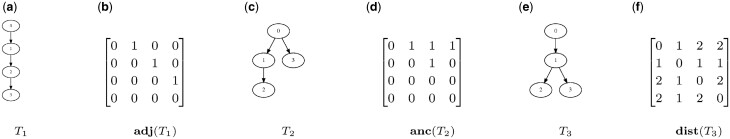
A couple of rooted trees and their matrices. (a) and (b) Mutation tree $T_{1}$ and its adjacency matrix. (c) and (d) Mutation tree $T_{2}$ and its ancestry matrix. (e) and (f) Mutation tree $T_{3}$ and its distance matrix.

Capital boldface letters (e.g. A) represent matrices. The element at the *i*th row and the *j*th column of a matrix A is denoted by A[i,j]. Given a graph *G*, the symbols V(G), E(G), and adj(G) stand for the vertex set, the edge list, and the adjacency matrix of *G*, respectively. In the sequel, all *n*-vertex graphs are vertex-labeled by the set $Z_{n}=\{0,\ldots ,n-1\}$ where we always assume that the rows and columns of any matrix assigned to *G* are ordered according to the natural order on $Z_{n}$ (see Fig. 1).

The distance between two vertices of a directed graph *G*, is the length of a shortest path between these vertices in an undirected sense. The “distance matrix” of *G*, denoted by dist(G), is defined as the matrix whose [*i*, *j*]th entry is the distance between the vertices *i* and *j*. Given a directed graph *G*, a vertex *v* is the “ancestor” of a vertex *u* (or *u* is the “descendent” of *v*) if there exists a directed path from *v* to *u* in *G*. By definition, the ancestor/descendant relationship is not reflexive. The “ancestry matrix” of *G*, denoted by anc(G), is defined as the binary matrix whose [*i*, *j*]th entry is 1 if the vertex *i* is the ancestor of the vertex *j*.

A “rooted tree” is a directed tree with a specific “root” vertex which is the ancestor of any other vertex. Note that directions in a rooted tree are by definition forced by the choice of the root. Because the labels in this context represent mutations, a single-labeled rooted tree is referred to as a “mutation tree.” The set of all *n*-vertex mutation trees is denoted by $T_{n}$.

In what follows $\varepsilon :T_{n}\to R^{n\times n}$ is an embedding of mutation trees into the space of real square matrices. We call a weight function $w:S\to R_{\geq 0}$ “normal” if $\sum_{s\in S}w(s)=1$. Also, let us recall the definition of the entry-wise $L_{p}$ matrix norms as:


$$\begin{array}{c} L_{p}(A):={\left(\sum_{i=1}^{n}\sum_{j=1}^{n}|A[i,j]{|}^{p}\right)}^{\frac{1}{p}},L_{\infty }(A):=\underset{0\leq i,j\leq n}{\max}|A[i,j]|. \end{array}$$

### 2.2 Dissimilarity measures

Given a mapping ε and a norm *L*,

$$\begin{array}{c} d_{\varepsilon ,L}(T_{1},T_{2}):=L(\varepsilon (T_{1})-\varepsilon (T_{2})) \end{array}$$

defines a dissimilarity measure on the trees through embedding. The injectivity of ε is a necessary and sufficient condition for $d_{\varepsilon ,L}$ to be a metric (see Supplementary Proposition 1).

Example 1. For the trees depicted in Fig. 1, we have

$$\begin{array}{c} d_{anc,L_{1}}(T_{1},T_{2})=L_{1}(anc(T_{1})-anc(T_{2}))=2. \end{array}$$

It can be verified that all dissimilarity measures defined with respect to adj, anc, and dist embeddings and $L_{1}$ and $L_{2}$ norms are computable in $O(n^{2})$ time (Supplementary Proposition 2). Interestingly, for the PCD, the ancestor–descendent distance, and the PD we have:


(1) $$\begin{array}{c} \text{PCD}(T_{1},T_{2})=d_{adj,L_{1}}(T_{1},T_{2})={\left(d_{adj,L_{2}}(T_{1},T_{2})\right)}^{2}, \end{array}$$

(2) $$\begin{array}{c} \text{ADD}(T_{1},T_{2})=d_{anc,L_{1}}(T_{1},T_{2})={\left(d_{anc,L_{2}}(T_{1},T_{2})\right)}^{2}, \end{array}$$

(3) $$\begin{array}{c} \text{PD}(T_{1},T_{2})=d_{d\mathrm{ist},L_{1}}(T_{1},T_{2}). \end{array}$$

#### 2.2.1 Metrics based on the distance matrix

As opposed to the adjacency matrix, which considers local relationships, the distance matrix incorporates long-distance relationships between vertices as well as local distances. Ancestry relations are not local, but their binary nature limits their power in expressing relations between vertices. Dissimilarity measures based on dist mapping assess whether labels that are close to (or far from) each other in one tree, are also close to (or far from) each other in the other tree.

Another perspective on $d_{d\mathrm{ist},L}$ is looking at bipartitions of the graph. The distance between two vertices *v* and *u* is actually the number of bipartitions of the tree in which these two vertices are in different partitions. Therefore, by comparing distance matrices of two trees, one is comparing a representation of their bipartitions.

### 2.3 The centroid tree problem

Consensus trees, already being studied in the literature, are best-fit trees defined as follows.

Definition 1 (The Weighted Consensus Tree Problem (CoTPP_*d*;*w*_)).

(Guang *et al.* (2023)) Let $d:T_{n}\times T_{n}\to R$ be a dissimilarity measure, set $S=\{T_{1},\ldots ,T_{k}\}\subset T_{n}$ be a set of mutation trees, and *w* a normal weight function on S. Then the “weighted consensus tree” of S, denoted by $T_{\text{con}}\in T_{n}$, is defined as:


$$\begin{array}{c} T_{\text{con}}(S):=\underset{T\in T_{n}}{\text{arg}\text{min}}\sum_{i=1}^{k}(w(T_{i})\cdot d(T_{i},T)). \end{array}$$

The case for the uniform weight function where the weights for all trees are equal to 1/k is denoted by ${\text{CoTP}}_{d}$.

GraPhyC (Govek *et al.* 2022) is a polynomial time algorithm to solve ${\text{CoTP}}_{\text{PCD}}$ and TuELiP (Guang *et al.* 2023) is an integer linear program (ILP) that solves ${\text{CoTP}}_{\text{ADD},w}$. Now, following the idea of embedding trees into an Euclidean space of matrices, we introduce a very general centroid tree as follows.

Definition 2 (The Weighted Centroid Tree Problem (CeTP_*ε*,*L*,*w*_)).

Let $\varepsilon :T_{n}\to R^{n\times n}$ be an injective embedding, $L:R^{n\times n}\to R$ be a norm. Given a set $S=\{T_{1},\ldots ,T_{k}\}\subset T_{n}$ of mutation trees and a weight function $w:S\to R_{\geq 0}$, the “weighted centroid tree” of S, denoted by $T^{*}\in T_{n}$, is defined as:


$$\begin{array}{c} T^{*}(S):=\underset{T\in T_{n}}{\text{arg}\text{min}}L(\sum_{i=1}^{k}(w(T_{i})\cdot \varepsilon (T_{i}))-\varepsilon (T)). \end{array}$$

The ω-“weighted centroid tree” problem denoted by $\omega {-\text{CeTP}}_{\varepsilon ,L}$ is the special case where *w* is the uniform weighting with all weights equal to $\frac{\omega }{k}$. Hereafter, the (classical) “centroid tree” problem is defined as $1{-\text{CeTP}}_{\varepsilon ,L}$ and is denoted by ${\text{CeTP}}_{\varepsilon ,L}$ in short.

The following example shows that CeTP and CoTP may have different solutions.

Example 2. Consider the trees $T_{1}$, $T_{2}$, and $T_{3}$ depicted in Fig. 1. Both $T_{1}$ and $T_{2}$ are optimal solutions of ${\text{CoTP}}_{d_{d\mathrm{ist},L_{1}}}$ for the set $S=\{T_{1},T_{2}\}$, having the cost $L_{1}(d\mathrm{ist}(T_{1})-d\mathrm{ist}(T_{2}))=8$. To see that $T_{3}$ is not an optimal consensus tree note that,

$$\begin{array}{c} L_{1}(d\mathrm{ist}(T_{1})-d\mathrm{ist}(T_{3}))+L_{1}(d\mathrm{ist}(T_{2})-d\mathrm{ist}(T_{3}))=6+6=12. \end{array}$$

On the other hand, $T_{3}$ is the single optimal solution of ${\text{CeTP}}_{d\mathrm{ist},L_{1}}$ having the cost


$$\begin{array}{c} L_{1}(\frac{d\mathrm{ist}(T_{1})+d\mathrm{ist}(T_{2})}{2}-d\mathrm{ist}(T_{3}))=2. \end{array}$$

None of $T_{1}$ and $T_{2}$ is an optimal centroid tree since for any T∈S,


$$\begin{array}{c} L_{1}(\frac{d\mathrm{ist}(T_{1})+d\mathrm{ist}(T_{2})}{2}-d\mathrm{ist}(T))=4. \end{array}$$

The next theorem demonstrates some cases when the solution sets of these optimization problems are equal.

Theorem 1. Given a set of mutation trees $S\subset T_{n}$ and a normal weight function *w* on S,

- for any binary embedding $\varepsilon :T_{n}\to {\left\{0,1\right\}}^{n\times n}$, the solution sets of ${\text{CeTP}}_{\varepsilon ,L_{1},w}$ and ${\text{CoTP}}_{d_{\varepsilon ,L_{1}},w}$ are identical.
- for any embedding $\varepsilon :T_{n}\to R^{n\times n}$, the solution sets of ${\text{CeTP}}_{\varepsilon ,L_{2},w}$ and ${\text{CoTP}}_{d_{\varepsilon ,L_{2}}^{2},w}$ are identical.

By the fact that both adj and anc are binary embeddings, one deduces that,

Corollary 1. The following sets of optimization problems admit the same set of solutions for any normal weight function *w*:

(a) {CoTP_PCD, *w*_, CeTP_adj, *L*_1_, *w*_, CeTP _adj, *L*_2_, *w*_},

(a) {CoTP_PCD, *w*_, CeTP_anc, *L*_1_, *w*_, CeTP _anc, *L*_2_, *w*_}.

### 2.4 The nearest mapped tree problem

Our main observation is the fact that the weighted centroid problem is an instance of the more general “nearest tree” problem. In the rest of this section, we will concentrate on this problem and provide some complexity results as well as some algorithms to solve it in some important cases.

Definition 3. (The Nearest Mapped Tree Problem (NMTP_*ε*,*L*_)). Let $\varepsilon :T_{n}\to R^{n\times n}$ be an embedding and $L:R^{n\times n}\to R$ be a norm. Given a matrix $M\in R^{n\times n}$, a nearest mapped tree $T^{*}(M)$ is defined as:

$$\begin{array}{c} T^{*}(M):=\underset{T\in T_{n}}{\text{arg}\text{min}}L(M-\varepsilon (T)). \end{array}$$

There are four ILPs and one integer quadratic program (IQP) introduced in this section to solve NMTP for ancestry and distance mappings (Table 1). We also provide different reductions from ${\text{NMTP}}_{adj,L_{2}}$ and ${\text{NMTP}}_{adj,L_{1}}$ to the maximum weighted arborescence (MWA) problem showing that both problems may be solved using algorithms for MWA. To prevent confusion, we use acronyms of the form “MapNorm” for the integer programs corresponding to ${\text{NMTP}}_{\text{Map},\text{Norm}}$ as well as their algorithmic solutions (e.g. as DistL1 for ${\text{NMTP}}_{d\mathrm{ist},L1}$). Also, we use the same acronym with a prefix determining the weight function for the algorithmic solutions of the corresponding weighted centroid problem. For instance, within this setting, our main contribution is to introduce the algorithm ω-AncL2 (Algorithm 2) to solve $\omega {-\text{CeTP}}_{anc,L_{2}}$.

**Table 1.** A list of algorithms and complexity results for different settings of CoTP, CeTP, and NMTP problems.^a^

ε	*L*	${\text{CoTP}}_{d_{\varepsilon ,L}}$	${\text{CeTP}}_{\varepsilon ,L}$	${\text{NMTP}}_{\varepsilon ,L}$
adj	$L_{1}$ and $L_{2}$	GraPhyC (Govek *et al.* 2022) P (Govek *et al.* 2022)	GraPhyC (Govek *et al.* 2022) P (Govek *et al.* 2022) (Corollary 1 (a))	Reductions to “Maximum Weight Arborescence” P (Theorem 2)
anc	$L_{1}$ and $L_{2}$	TuELiP (Guang *et al.* 2023) NP-hard (Qi and El-Kebir 2024)	TuELiP (Guang *et al.* 2023) NP-hard (Qi and El-Kebir 2024) (Corollary 1 (b))	AncL1 for $L_{1}$ AncL2 for $L_{2}$ NP-hard (Corollary 2)
dist	$L_{1}$	No algorithm yet Complexity unknown	DistL1 Complexity unknown	DistL1 NP-hard (Theorem 3)
dist	$L_{2}$	DistL2 Complexity unknown (Theorem 1 (b))	DistL2 Complexity unknown	DistL2 NP-hard (Theorem 3)
dist	$L_{\infty }$	No algorithm yet Complexity unknown	DistLInf Complexity unknown	DistLInf Complexity unknown

a The algorithms in blue are presented in this paper. The set P denotes the set of polynomial time solvable problems.

Theorem 2. The problems ${\text{NMTP}}_{adj,L_{1}}$ and ${\text{NMTP}}_{adj,L_{2}}$ can be solved in $O(n^{3})$ time through finding a maximum weight spanning arborescence tree.

Note that although ${\text{NMTP}}_{adj,L_{1}}$ and ${\text{NMTP}}_{adj,L_{2}}$ are reduced to the MWA problem, their solutions may be different as a consequence of using different reductions.

For the ancestry matrix embedding, recently, Qi and El-Kebir (2024) proved that ${\text{CoTP}}_{\text{ADD}}$ is an NP-hard problem. Hence, Corollary 1 implies that ${\text{NMTP}}_{anc,L}$ is also NP-hard.

Corollary 2. The problems ${\text{CeTP}}_{anc,L_{1}}$, ${\text{CeTP}}_{anc,L_{2}}$, ${\text{NMTP}}_{anc,L_{1}}$, and ${\text{NMTP}}_{anc,L_{2}}$ are NP-hard.

On the algorithmic side, we developed two ILPs solving ${\text{NMTP}}_{anc,L_{1}}$ and ${\text{NMTP}}_{anc,L_{2}}$, namely AncL1 (Supplementary Algorithm 2) and AncL2 (Supplementary Algorithm 1), respectively. These ILPs are similar to the one provided for TuELiP, but with different cost functions and some differences in constraints.

Algorithm 1. The ILP program AncL2 that solves ${\text{NMTP}}_{anc,L_{2}}$. The input is the matrix M having $m_{ij}$ as its entries. For an optimal solution for this ILP, the matrix X, having $x_{ij}$s as entries, is anc(T) for a tree $T\in T_{n}$.

$\begin{array}{ccc} \text{minimize} & \sum_{0\leq i,j\leq n}\left(x_{ij}-2m_{ij}x_{ij}\right) & \\ \text{subject to} & x_{ij}+x_{jk}-x_{ik}\leq 1 & \forall i,j,k\in Z_{n}\\ & x_{ij}+x_{ji}\leq 1 & \forall i,j\in Z_{n}\\ & x_{ik}+x_{jk}-x_{ij}-x_{ji}\leq 1 & \forall i,j,k:i\neq j\neq k\neq i\\ & \sum_{j\neq i}x_{ij}=y_{i} & \forall i\in Z_{n}\\ & \sum_{0\leq i\leq n}z_{i}=1 & \\ & y_{i}\geq (n-1)z_{i} & \forall i\in Z_{n}\\ & y_{i}\leq (n-2)(1+z_{i}) & \forall i\in Z_{n}\\ & x_{ij},z_{i}\in \{0,1\} & \forall i,j\in Z_{n}\\ & 0\leq y_{i}\leq n-1 & \forall i\in Z_{n} \end{array}$

For the distance matrix embedding, we first present the following hardness result.

Theorem 3. The problems ${\text{NMTP}}_{d\mathrm{ist},L_{1}}$ and ${\text{NMTP}}_{d\mathrm{ist},L_{2}}$ are NP-hard.

It must be noted that, to the best of our knowledge, computational complexities of the problems ${\text{NMTP}}_{d\mathrm{ist},L_{\infty }}$, ${\text{CeTP}}_{d\mathrm{ist},L_{1}}$, ${\text{CeTP}}_{d\mathrm{ist},L_{2}}$, and ${\text{CeTP}}_{d\mathrm{ist},L_{\infty }}$ are not settled yet. On the algorithmic side, we provide two ILPs DistL1 (Supplementary Algorithm 3) and DistLInf (Supplementary Algorithm 5) and an IQP DistL2 (Supplementary Algorithm 4) to solve the problems ${\text{NMTP}}_{d\mathrm{ist},L_{1}}$, ${\text{NMTP}}_{d\mathrm{ist},L_{\infty }}$, and ${\text{NMTP}}_{d\mathrm{ist},L_{2}}$, respectively.

Algorithm 2.
ω-AncL2

**Input:** A set of mutation trees $S=\{T_{1},\ldots ,T_{k}\}\subset T_{n}$ and a weight ω

**Output:** A single tree $T\subset T_{n}$

$$M\leftarrow \frac{\omega }{k}\sum_{i=1}^{k}anc(T_{i})$$

Solve AncL2 for input M and **return** the output.

### 2.5 A Second look at weighted centroids

In Section 2.3, we explored the close relationship between weighted consensus trees and weighted centroid trees for normal weight functions. In this section, we are going to explore the effect of weight functions when they are not normalized and show that this, in particular, may be considered as an advantage in some cases of interest.

It is straightforward to verify that nonnormal weight functions do not change the solution set of the weighted consensus tree problem, however, on the other hand, from the perspective of the NMTP, using nonnormal weight functions makes it possible to shift the centroid point of a set of trees arbitrarily away from the classical centroid tree of the set, which may give rise to a significantly different centroid tree, in general.

The TuELiP algorithm demonstrates that different weights for input trees may improve the quality of the summarization method. To reduce the complexity of analysis and demonstrate the effectiveness of using nonnormal weight functions, in what follows, we just concentrate on the case of uniform nonnormal weight functions (i.e. the case of ω-weighted centroids) that may be interpreted as scaling the classical centroid point.

To begin, one may easily verify that the maximum weight spanning arborescence tree does not change by scaling, implying that for the case of adjacency matrix embedding, the weighted setup will not provide any new information. In other words,

Corollary 3. For any ω>0 the solutions to the problems ${\text{CeTP}}_{adj,L_{2}}$ and $\omega {-\text{CeTP}}_{adj,L_{2}}$ are identical.

Interestingly, for the ancestry matrices the story is completely different. Note that for the case of ancestry relations, one may argue that the signals are asymmetric, in the sense that the “presence” of ancestry signals is more informative than their “absence.” For instance, if mutation $m_{1}$ is observed as an ancestor of mutation $m_{2}$ in 49 out of 100 patients, this is a strong indication that $m_{1}$ is an ancestor of $m_{2}$. Hence, in this case, $M_{c}[m_{1},m_{2}]=0.49$ for the centroid point, while the absence of an ancestry relation is preferred when solving the centroid tree problem. However, this intuition is not in coherence with the equal treatment of presence and absence of ancestry relations by ${\text{CeTP}}_{anc,L}$.

Scaling the centroid point is a way to increase the strength of ancestry signals. Of course, one may solve $\omega {-\text{CeTP}}_{anc,L_{2}}$ for some candidate values of ω>1 using the ω-AncL2 algorithm. According to our example, having 49 observations of $m_{1}$ as an ancestor of $m_{2}$, one may verify that for ω=2 the new value for $M_{c}[m_{1},m_{2}]$ is 0.98 which is quite close to 1. We have tried ω=2 and ω=3 in our analysis, where the results show that ω=2 seems to be a better uniform choice for the weight function. It must be mentioned that the best choice for ω may not be an integer value and developing methods to determine a close to optimal value for ω is of practical importance and remains to be studied as an independent topic.

Moreover, it must be noted that scaling the centroid for distance matrix does not follow the same intuition as for the ancestry matrix and cannot be interpreted as strengthening the signals.

## 3 Experimental results

Algorithms and simulations are implemented on Python (3.7.9). ILPs are solved by IBM ILOG CPLEX Optimizer (12.9) with DOcplex (2.23.222) python API (details in Supplementary Section 5).

### 3.1 Synthetic-data generation

To synthesize the data, first, a set of random labeled rooted trees are generated as the ground truth trees. All generated trees are rooted at 0 depicting germ-line cells. Then, we generate a consistent cancer cell fractions (according to the sum rule; Jiao *et al.* 2014) randomly in a top-down manner. From a single ground truth tree, a set of altered trees are generated by randomly applying the following alterations: (i) Swap a child vertex with its parent (parent–child: **pc**), (ii) Move a vertex *v* with all of its descendants to become a child of another vertex *p* (branch-move: **bm**), and (iii) Remove a vertex from where it is to become one of the children of the root (node-remove: **nr**).

The simulation parameter, **cp** (change-probability), determines the number of changes applied to each tree. The alteration **bm** is already applied in simulations reported for ConTreeDP (Fu and Schwartz 2021) and TuELiP (Guang *et al.* 2023) and the **pc** alteration is introduced as an alternative to “edge collapse” modification in mentioned contributions, since we only consider single-labeled trees. In CeTP, it is assumed that all input trees have the same set of labels (mutations). If some mutations are absent in a number of trees, they can be added as direct children of the root vertex in those trees. This may be considered as the main motivation to add the node-remove alteration to our simulation setup. The noise observed in detection of mutations in certain phylogenies can be attributed to various sources. Biological sources include intra- and intertumor heterogeneity, while technological sources encompass sequencing errors and depth (details of simulation method appears in Supplementary Section 6).

### 3.2 CeTP typically has a unique solution

Note that CeTP does not necessarily admit a unique solution. In this section, we study the number of optimal solutions of the optimization problems ${\text{CeTP}}_{anc,L_{2}}$, ${\text{CeTP}}_{d\mathrm{ist},L_{1}}$, ${\text{CeTP}}_{d\mathrm{ist},L_{2}}$ and ${\text{CeTP}}_{d\mathrm{ist},L_{\infty }}$. To find the number of optimal solutions, we enumerate all trees having the same label set with n=7 vertices. We have reported the number of optimal solutions for k=3,10,15 in 100 instances of the problem for each *k*.

**Figure 2.**
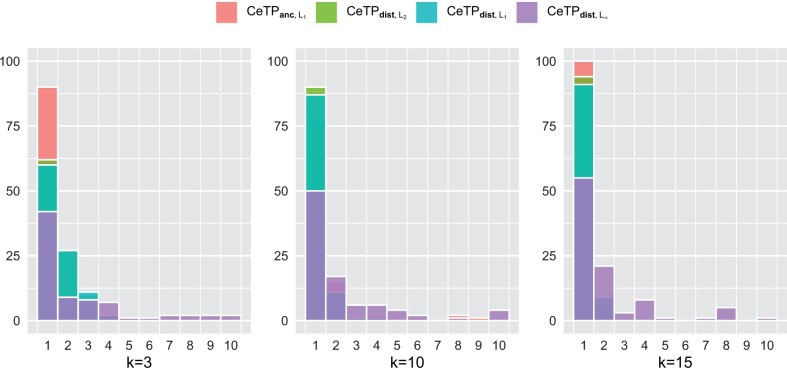
Overlaying histograms of the number of optimal solutions for 100 instances of ${\text{CeTP}}_{anc,L_{2}}$, ${\text{CeTP}}_{d\mathrm{ist},L_{1}}$, ${\text{CeTP}}_{d\mathrm{ist},L_{2}}$, and ${\text{CeTP}}_{d\mathrm{ist},L_{\infty }}$ on trees with seven vertices for *k *=* *3, *k *=* *10, and *k *=* *15 input trees.

${\text{CeTP}}_{d\mathrm{ist},L_{\infty }}$ has significantly more solutions per instance than the other three problems (Fig. 2), which seems to be natural since the $d_{d\mathrm{ist},L_{\infty }}$ norm has a maximum peak that may allow many small variations in the structures of optimal trees with the same cost. On the other hand, ${\text{CeTP}}_{anc,L_{2}}$, ${\text{CeTP}}_{d\mathrm{ist},L_{1}}$ and ${\text{CeTP}}_{d\mathrm{ist},L_{2}}$ usually have unique solutions, with some sporadic exceptions for which the multiplicity of solutions rarely goes above two (Fig. 2). It is also interesting to note that the number of optimal solutions decreases as the number of input trees increases (Fig. 2).

### 3.3 Weighting helps to prevent loss of the ancestry signals

When solving ${\text{CeTP}}_{anc,L_{2}}$ with 1-AncL2, it should be pointed out that many optimal solution trees are star-shaped, indicating that all ancestry relations are lost except for the germ-line cell, which is the ancestor of all vertices in all trees by construction. On the other hand, by increasing the weight of the ancestry relations, it is expected to have a decrease in the number of star-shaped solutions. We observed that for our 300 simulations on trees with seven vertices and k=3,10,15, there are 233, 34, and five star-shaped trees for solutions of 1-AncL2, 2-AncL2, and 3-AncL2, respectively.

### 3.4 Node-remove alterations produce harder instances to solve

**Figure 3.**
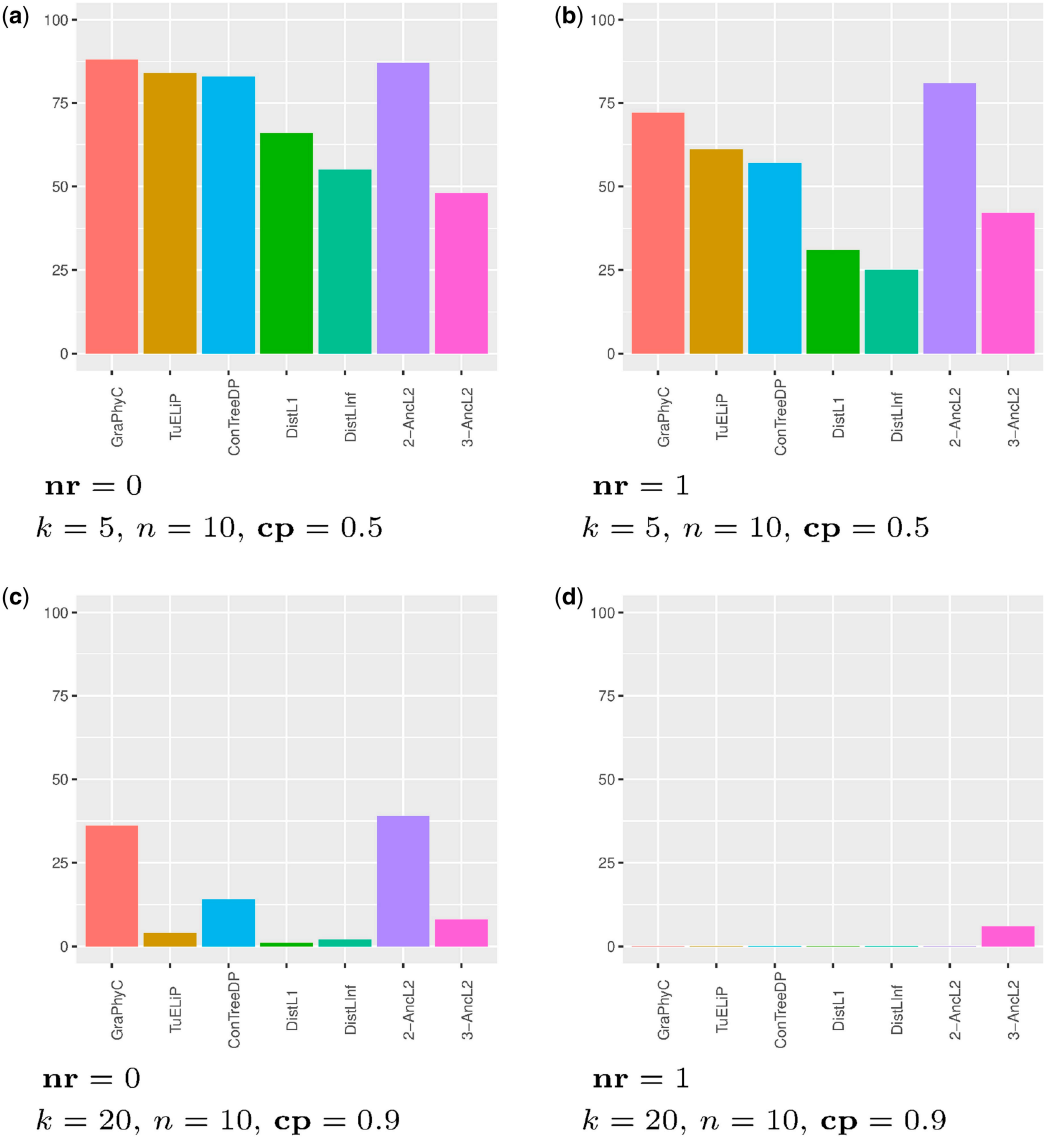
The effect of node-remove alteration in hardness of finding the ground truth tree. The plots show the number of trials in which different algorithms output the ground truth tree with respect to, including or excluding, node-remove alterations in the simulation process for two simple simulation settings (a, b) and two hard ones (c, d). Total number of simulations in each setting is 100. Other simulation parameters are pc=1, bm=1.

It is interesting to report our observation that introduction of node-remove alterations significantly increases the complexity of finding the ground truth tree for all the algorithms we applied. Each algorithm actually finds fewer ground truth trees when the **nr** alteration is incorporated into the simulations (Fig. 3). For instance, the two best-performing algorithms in Fig. 3c are 2-AncL2 and GraPhyC. Considering 100 simulations, 2-AncL2 and GraPhyC identify 39 and 35 true trees, respectively, when no node-remove change is applied to the dataset; however, when the node-remove alteration is applied in simulations, none of these two algorithms is able to recover even a single ground truth tree. Based on arguments for validity of the node-remove alteration mentioned before (Section 3.1), this fact, in our opinion, introduces more challenging algorithm design problems to be considered in this field of research.

### 3.5 Benchmarking the algorithms

We evaluate the algorithms by comparing the distance between their outputs and the ground truth tree for the input set. We use n=10,20 vertices and $k=\frac{n}{2},n,2n$ input trees for simulations, with at least one of the three alteration methods (**pc**, **bm**, and **nr**) active in each simulation setting. This produces 2×3×7 simulation settings, with 100 tree sets generated for each setting.

We have used CASet, DISC, PCD, ADD, $d_{d\mathrm{ist},L_{1}}$, $d_{d\mathrm{ist},L_{2}}^{2}$, and $d_{d\mathrm{ist},L_{\infty }}$ dissimilarity measures to compare the distance between algorithm outputs and the ground truth tree. To evaluate our algorithms (DistL1, DistLInf, 2-AncL2, and 3-AncL2), we compare the results with three state-of-the-art algorithms: (i) GraPhyC, (ii) TuELiP, and (iii) ConTreeDP. Notably, TuELiP is applied in its uniformly weighted version because there is no basis for nonuniform weighting of trees in our simulation process.

All algorithms perform similarly according to all distance measures, except for PCD and $d_{d\mathrm{ist},L_{\infty }}$ (see Supplementary Fig. S5). We have also selected the CASet distance, previously used in evaluations of ConTreeDP and TuELiP, as our comparison measure. This measure is defined as the size of the symmetric difference between the sets of common ancestors for all pairs of mutations in the trees. We believe that this will establish a unified framework, enabling a fair comparison of performance measures among the proposed algorithms.

**Figure 4.**
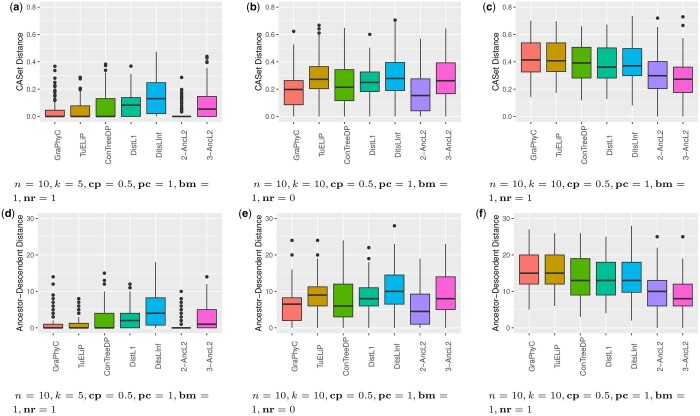
The CASet and AD distance between output of the algorithms and the ground truth tree for three different simulation settings. The *x*-axis shows different algorithms and the *y*-axis shows the distance between output of the algorithm and the ground truth tree.

One may observe that 2-AncL2 is always the best or in the worst case the second-best algorithm in all simulation settings (see Table 2, Fig. 4, and Supplementary Figs S6 and S7). When the **nr** alteration is present, 3-AncL2 performs better than all other algorithms, with 2-AncL2 being the second-best. In the absence of **nr** alterations, 2-AncL2 and GraPhyC are two of the best-performing algorithms. Also, 2-AncL2 is better than TuELiP in all simulation settings except when only **pc** alterations are applied (Supplementary Table S5). This in our opinion, is a concrete evidence that the idea of using the weighted setup for the ancestry signal improves the performance of TuELiPs in 2-AncL2.

**Table 2.** The best and the second best algorithms having the smallest average CASet distance (the number in the parenthesis) to the ground truth trees for different simulation settings.^a^

**pc, bm, nr** ^b^	n=20 , k=10	n=20 ,k=20	n=20 ,k=40
** 1, 1, 0**	1-GraPhyC(0.06)	1-GraPhyC(0.02)	1-GraPhyC(0.00)
	1-2-AncL2(0.06)	1-2-AncL2(0.04)	1-2-AncL2(0.02)
	2-ConTreeDP(0.13)	2-ConTreeDP(0.11)	2-ConTreeDP(0.07)
** 1, 0, 0**	1-ConTreeDP(0.00)	1-GraPhyC(0.00)	1-GraPhyc(0.00)
	2-GraPhyC(0.01)	1-TuELiP(0.00)	1-TuELiP(0.00)
	2-TuELiP(0.01)	1-ConTreeDP(0.00)	1-2-AncL2(0.00)
	2-2-AncL2(0.02)	1-2-AncL2(0.00)	1-ConTreeDP(0.00)
** 0, 1, 0**	1-GraPhyC(0.00)	1-GraPhyC(0.00)	1-GraPhyC(0.00)
	2-2-AncL2(0.04)	2-2-AncL2(0.03)	2-2-AncL2(0.02)
0 or 1,0 or 1,1	1-3-AncL2(0.12)	1-3-AncL2(0.07)	1-3-AncL2(0.04)
	2-2-AncL2(0.16)	2-2-AncL2(0.15)	2-2-AncL2(0.13)

a Algorithms in bold are introduced in this article (details in Supplementary Section 7).

b Parent–child, branch-move, and node-remove parameters for simulation method. The numbers 1/0 represent “including” or “excluding” the corresponding alteration, respectively. In the last row, the distance is reported for pc=bm=nr=1.

On the other hand, DistL1 performs quite similar to the ConTreeDP algorithm (Supplementary Table S6), where just five out of 21 settings show significant difference in the mean distance to the ground truth trees for the two algorithms.

We have also observed that the evaluation process of the algorithms significantly depends on the simulation methods used. For instance, it has been reported in Fu and Schwartz (2021) that ConTreeDP outperforms GraPhyC in almost every simulation set (see Supplementary Fig. S3). Conversely, the dataset used to evaluate TuELiP in Guang *et al.* (2023) shows that GraPhyC and TuELiP outperform ConTreeDP in almost all simulation settings (Guang *et al.* 2023). Our primary objective was to establish a simulation setup capable of encompassing potential natural alterations, while also ensuring robustness and flexibility, distinguishing it from previous setups.

### 3.6 Experiments on real data

We applied our methods to a real data of breast cancer provided by the cohort study of Razavi *et al.* (2018). The dataset comprises targeted sequencing of 1918 tumors from 1756 breast cancer patients, focusing on protein-coding exons of genes that are associated with cancer. We used the tree sets obtained by applying SPRUCE (El-Kebir *et al.* 2016) algorithm on this dataset for patients with SNVs occurring in copy neutral autosomal regions. SPRUCE enumerates all tumor phylogenies that explain the variant allele frequencies of the SNVs (data available on GitHub page of the RECAP algorithm; Christensen *et al.* 2020).

**Figure 5.**
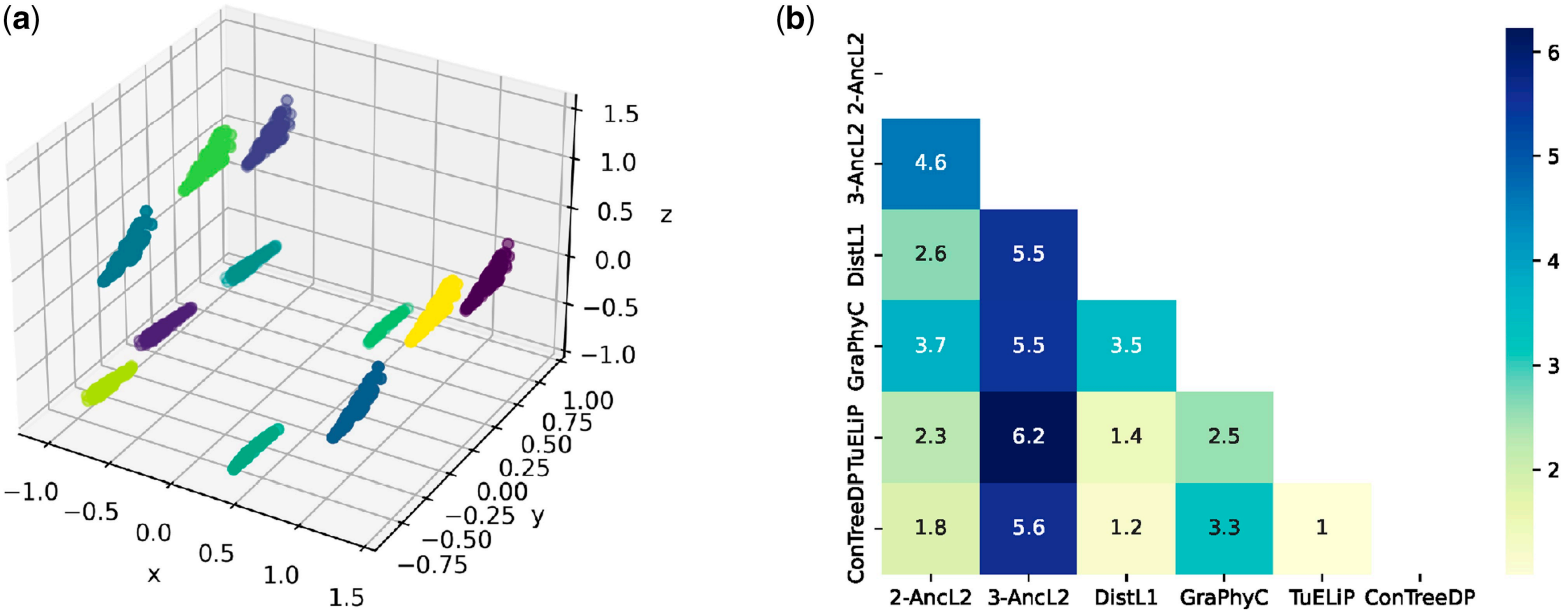
(a) 3D PCA for adjacency matrices of 970 trees for patient P-0014476. (b) Sum of CASet distances between centroid trees (of clusters for patient P-0014476) obtained by different summarization methods for every pair of methods.

For our analysis, we selected patient P-0014476, for whom there are 970 trees with nine vertices compatible with the patient’s data obtained from SPRUCE. Naturally, a systematic approach is required to study all these trees. We first applied PCA (principal component analysis) to the adjacency matrices of the trees. This reduced the dimensions from 9 to 3, enabling us to visualize all 970 trees. We observed the 11 visible clusters for patient P-0014476 in three dimensions (see Fig. 5a) by applying K-means clustering. The cluster statistics reveal a minimum size of 46, a maximum size of 103, and an average size of 89.

In a second stage, the algorithms 2-AncL2, 3-AncL2, DistL1, TuELiP, GraPhyC, and ConTreeDP are applied to obtain a summarization consisting of 11 trees (see Supplementary Figs S8–S13). The two most similar methods are TuELiP and ConTreeDP, however, all clusters have different centroid trees obtained from these two methods (Fig. 5b). Also, 2-AncL2s centroid trees are similar to ConTreeDPs, with four clusters (cl0, cl1, cl7, cl8) having the same centroid tree for both methods. It ought to be noted that 3-AncL2 produces the deepest trees, while TuELip produces the shallowest.

Next, we considered centroid trees resulted by 2-AncL2 algorithm. We observed that NOTCH1 gene is mutated earliest in centroid trees for all clusters using 2-AncL2 (Supplementary Fig. S8). The prognostic and regulatory role of Notch signaling pathway in breast cancer have been recently addressed (Cravero *et al.* 2022, Yousefi *et al.* 2022). Notch1 signaling encompasses a wide range of functions in determining cell fate, cell proliferation, and apoptosis (Artavanis-Tsakonas 1988, Leong and Karsan 2006). The NOTCH1 gene has been associated with both oncogenic and tumor suppressing roles (Lobry *et al.* 2011).

In all centroid trees, NOTCH1 is directly followed by a subset of four genes: ALOX12B, TP53, MAP3K1, and XPO1. This finding may suggest an early event in carcinogenesis and potentially intimate the causal effects of these mutations in breast cancer. It is noteworthy that mutations in both TP53 and MAP3K1 have been demonstrated to significantly contribute to the pathogenesis of breast cancer (Pham *et al.* 2013, Huszno and Grzybowska 2018).

ALOX12B is a gene encoding a lipoxygenase enzyme, and is responsible for the conversion of arachidonic acid to 12-HETE (Krieg *et al.* 2001). Lipoxygenases are reported to be associated with tumorigenesis with a mutation in ALOX12B has already been detected in lung (Shen *et al.* 2009) and cervical cancers (Jiang *et al.* 2020). However, the role of ALOX12B in breast cancer is not well established and limited studies have shown its role in this cancer (Lee *et al.* 2009, Rooney *et al.* 2015).

XPO1 is a gene that encodes an export receptor responsible for the nuclear-cytoplasmic transportation of hundreds of proteins and multiple RNA species. It is known that this gene is frequently overexpressed and/or mutated in some human cancers (including breast cancer) and plays role as a driver oncogene, making it a good target for breast cancer treatment (Azizian and Li 2020, Kim *et al.* 2022, Landes *et al.* 2023). Further research is needed to determine the potential role of ALOX12B and NOTCH1 in development and progression of breast cancer.

An alternative method for clustering this data involves the MCT problem (Aguse *et al.* 2019). We examined the clusters resulting from MCT with k = 11 clusters. Three algorithms for solving the MCT problem are presented in Aguse *et al.* (2019): two exact algorithms (a brute force and an ILP) and a heuristic one (coordinate ascent). The heuristic algorithm is designed to solve large instances of the problem, and our set of 970 trees is three times larger than the largest example in Aguse *et al.* (2019). Therefore, we utilized the output of the coordinate ascent algorithm with 2000 random restarts. The clustering obtained closely resembles our clustering, with an adjusted rand index of 0.93. Notably, our clustering yields a lower cost with respect to the objective function of the MCT problem. More specifically, the cost function is the sum of the PCDs of trees in clusters with the centroids of the same clusters. The cost of our clustering is 3269.8, while the cost of the output clustering from the coordinate ascent algorithm is 3352.6. This demonstrates the time efficiency of our method and its accuracy in identifying clusters within the input tree set.

## 4 Concluding remarks

We proposed the concept of “weighted centroid trees” to provide a flexible setup for summarizing and analyzing classes of mutation trees. Within this framework one is free to choose the embedding map as well as the norm to tune one’s best options based on the problem at hand, however, as we have discussed and proved through this contribution, such choices have deep impacts on the solvability of the problem itself. Considering our experimental results, we proposed a new algorithm ω-AncL2, based on the variant choosing the ancestry matrix for the embedding and the $L_{2}$ norm to set the distance, that has a superior performance in our experiments among the rest of the algorithms available at the time of writing this article. In this regard, we would like to mention some concluding remarks as follows.

Finding efficient methods to determine the best choice for the weight function in the presented setup, in particular, for the ω-AncL2 algorithm, is among the interesting problems that ought to be studied in future research.

In this study, we have confined our theoretical analysis to single-labeled trees as an initial step to define and analyze the centroid tree problem. Although from a practical perspective, our experimental results show the effectiveness of this approach, we have also benchmarked the performance of TuELiP and its single-labeled counterpart in our setup, namely (AncL2), on the two originally provided synthetic multilabeled datasets in Fu and Schwartz (2021); Guang *et al.* (2023), showing that the performance of these algorithms are essentially the same, justifying validity and efficiency of our setup (see Supplementary Section 8).

Generalizations of our theoretical results to the case of multilabeled trees is a subject for further research. It also must be noted that there exist some other contributions in current literature on phylogenetic trees that try to use non-Euclidean metric structures to improve existing classical Euclidean methods and results (e.g. see Monod *et al.* (2018) for embeddings into hyperbolic spaces and Matsumoto *et al.* (2021) for using tropical spaces and ultrametrics). Although these approaches are different from what we have pursued in this article, possible extensions of our framework to non-Euclidean setups are another aspect that ought to be studied in future research.

## Supplementary Material

btae120_Supplementary_Data

## Data Availability

The data underlying this article are available in GitHub, at https://github.com/vasei/WAncILP.
