# Supplementary material for: Weighted centroid trees: a general approach to summarize phylogenies in single-labeled tumor mutation tree inference
Source: Bioinformatics. 2024 Jul 10;40(7):btae120. doi: 10.1093/bioinformatics/btae120 (PMC11520232; doi:10.1093/bioinformatics/btae120)
Supplement: btae120_Supplementary_Data [file btae120_supplementary_data.pdf]

Supplementary Material - Weighted Centroid Trees: A general approach to summarize phylogenies in single-labeled tumor mutation tree inference

Hamed Vasei
Mohammad-Hadi Foroughmand-Araabi
Amir Daneshgar

February 2024

Contents

1 Notations **5**

2 Dissimilarity measures **5**
2.1 PCD and ADD . . . 6

3 Consensus vs Centroid Tree **7**

4 Nearest Mapped Tree **9**
4.1 $\text{NMTP}_{\mathbf{adj},L}$ . . . 9
4.2 $\text{NMTP}_{\mathbf{anc},L}$ . . . 11
4.3 $\text{NMTP}_{\mathbf{dist},L}$ . . . 14
4.3.1 $\mathcal{NP}$-hardness . . . 14
4.3.2 ILPs . . . 18

5 Implementation Details **20**

6 Simulation Method **21**

7 Ranking Table **23**

8 Single-labeled AncL2 vs Multi-labeled TuELiP **24**

9 Supplementary images and tables **28**

10 Acknowledgement **38**

List of Theorems

1 Proposition . . . 5
2 Proposition . . . 5
1 Theorem . . . 9
2 Theorem . . . 9
3 Theorem . . . 14

List of Algorithms

1 AncL2 . . . 12
2 AncL1 . . . 14
3 DistL1 . . . 19
4 DistL2 . . . 20
5 DistLInf . . . 21

List of Figures

1 Comparing TuELiP to ConTreeDP and GraPhyC from the [GSO23]. 25
2 Number of found ground truth trees [GSO23]. 26
3 Results of running different algorithms on ConTreeDP original simulated data. Clearly, ConTreeDP has the best performance on this dataset. It identifies the true structure of most of the trees. 28
4 PCA visualization for two patients using **adj**, **anc**, and **dist** mappings. Almost zero adjusted rand index shows low concordance between clusters of different mappings (table 4) 28
5 Pearson correlation between different distance measures for solutions of the same algorithm in various simulation settings. Each block shows Pearson correlation between different distance measures in computing the distance between solution trees and the ground truth trees for 100 samples of one specific mutation settings solved by a specific algorithm. 29
6 Distance to ground truth tree of outputs of different algorithms in different simulation settings on trees with 10 nodes. A single simulation setting with different distance measures are depicted in each row. Each block shows the box plot of distances between the solutions of different algorithms and the ground truth trees for 100 instances of a specific simulation settings computed by a specific distance measure. 30
7 Distance to ground truth tree of outputs of ancestry based algorithms in different simulation settings on trees with 10 or 20 nodes. A single simulation setting with different distance measures are depicted in each row. Each block shows the box plot of distances between the solutions of different algorithms and the ground truth trees for 100 instances of a specific simulation settings computed by a specific distance measure. 31
8 Centroid trees for 11 clusters of adjacency matrices for patient P-0014476 using 2-AncL2 algorithm. 32
9 Centroid trees for 11 clusters of adjacency matrices for patient P-0014476 using 3-AncL2 algorithm. 33
10 Centroid trees for 11 clusters of adjacency matrices for patient P-0014476 using DistL1 algorithm. 34
11 Centroid trees for 11 clusters of adjacency matrices for patient P-0014476 using GraPhyC algorithm. 35
12 Centroid trees for 11 clusters of adjacency matrices for patient P-0014476 using TuELiP algorithm. 36
13 Centroid trees for 11 clusters of adjacency matrices for patient P-0014476 using ConTreeDP algorithm. 37

List of Tables

1 Best performing algorithms in each simulation setting. First and second algorithms with smallest average CASet distance (the number in the parenthesis) to the ground truth trees are reported for different simulation settings. . . . 23

2 Comparison between mean CASet distance of 100 instances for each simulation settings between TuELiP and its single-label counterpart AncL2 algorithms on ConTreeDP original simulated data. The p-value is obtained from a paired t-test. There is no significant difference in the mean of distances with a p-value threshold of 0.05. 25

3 Comparison between average CASet distance for 100 instances in 9 simulation scenarios between outputs of different methods and the ground truth trees. 27

4 Rand index and adjusted rand index for comparing cluster agreement between clusters of patient P-0000041's trees using adjacency, ancestry and distance mappings. Near zero adjusted rand index suggests that these clusters does not agree with each other more than any two random clustering of data with this sizes. 38

5 Comparison between mean CASet distance of 100 instances for each simulation settings between TuELiP and 2-AncL2 algorithms. The p-value is obtained from a paired t-test. The threshold for p-values significance is 10^{-3}. The blue color shows smaller mean distance value (red is the opposite). The yellow color specifies insignificant p-values. The dominance of blue value in the 2-AncL2 column, is an evidence for its better performance. 39

6 Comparison between mean CASet distance of 100 instances for each simulation settings between ConTreeDP and DistL1 algorithms. The p-value is obtained from a paired t-test and rounded to 3 decimal points. The threshold for p-values significance is 10^{-2}. The yellow color specifies insignificant p-values (green is the opposite). Dominance of yellow color suggests that these two algorithms have comparable performance. 40

1 Notations

In addition to notations already defined in the main text, in the sequel vectors are denoted by lowercase boldface letters (like $\mathbf{a}$). Also,, the *inner product* of two matrices is defined as

$$\langle A, B\rangle := \sum_{i,j} A[i,j]B[i,j].$$

Note that for any real number r we have

$$r\langle A, B\rangle = \langle rA, B\rangle = \langle A, rB\rangle.$$

2 Dissimilarity measures

Proposition 1. The dissimilarity measure $\mathrm{d}_{\varepsilon,L}$ is a metric *if and only if* $\varepsilon\colon \mathcal{T}_n \to \mathbb{R}^{n\times n}$ is one-to-one.

Proof. If ε is not one-to-one then there exist two distinct trees T_1 and T_2 for which $\varepsilon(T_1) = \varepsilon(T_2)$, hence $\mathrm{d}_{\varepsilon,L}(T_1, T_2) = 0$ and $\mathrm{d}_{\varepsilon,L}$ is not a metric. Conversely, *if* ε is one-to-one then $\mathrm{d}_{\varepsilon,L}$ is a metric:

1. The distance from a point to itself is zero, since $\mathrm{d}_{\varepsilon,L}(T_1, T_1) = L(0) = 0$.
2. (Positivity) The distance between two distinct points is always positive, since if $T_1 \neq T_2$, then $\varepsilon(T_1) \neq \varepsilon(T_2)$, implying $x = \varepsilon(T_1) - \varepsilon(T_2) \neq 0$, and consequently, $\mathrm{d}_{\varepsilon,L}(T_1, T_2) = L(x) \neq 0$.
3. (Symmetry) The distance from T_1 to T_2 is the same as the distance from T_2 to T_1, since $\mathrm{d}_{\varepsilon,L}(T_1, T_2) = L(\varepsilon(T_1) - \varepsilon(T_2)) = L(\varepsilon(T_2) - \varepsilon(T_1)) = \mathrm{d}_{\varepsilon,L}(T_1, T_2)$.
4. The triangle inequality holds, since $\mathrm{d}_{\varepsilon,L}(T_x, T_z) = L(\varepsilon(T_x) - \varepsilon(T_z)) = L(\varepsilon(T_x) - \varepsilon(T_y) + \varepsilon(T_y) - \varepsilon(T_z)) \leq L(\varepsilon(T_x) - \varepsilon(T_y)) + L(\varepsilon(T_y) - \varepsilon(T_z)) = \mathrm{d}_{\varepsilon,L}(T_x, T_y) + \mathrm{d}_{\varepsilon,L}(T_y, T_z)$.

□

Proposition 2. All dissimilarity measures $\mathrm{d}_{\mathbf{adj},L_1}$, $\mathrm{d}^2_{\mathbf{adj},L_2}$, $\mathrm{d}_{\mathbf{anc},L_1}$, $\mathrm{d}^2_{\mathbf{anc},L_2}$, $\mathrm{d}_{\mathbf{dist},L_1}$, $\mathrm{d}^2_{\mathbf{dist},L_2}$ and $\mathrm{d}_{\mathbf{dist},L_\infty}$ are computable in $\mathcal{O}(n^2)$ time.

Proof. All three matrices for adjacency, ancestry, and distance can be computed in $\mathcal{O}(n^2)$ time, by running a BFS from the root towards the leaves and updating $\mathcal{O}(n)$ entries at each round. Comparing two matrices element-wise is also possible in $\mathcal{O}(n^2)$ time. □

2.1 PCD and ADD

The binary operator $\oplus$ is the *symmetric difference* operator.

Definition 1 (PCD)**.** ([Gov+20]) The *parent-child set* of a tree T is defined as

$$\phi_{PC}(T) \coloneqq \{(i,j) \mid i \text{ is the parent of } j \text{ in } T\}.$$

Given two mutation trees T_1 and T_2, the *parent-child distance* is defined as

$$\text{PCD}(T_1, T_2) \coloneqq |\, \phi_{PC}(T_1) \oplus \phi_{PC}(T_2)|.$$

Definition 2 (ADD)**.** ([Gov+20]) The *ancestor-descendent set* of a tree T is defined as

$$\phi_{AD}(T) \coloneqq \{(i,j) \mid i \text{ is an ancestor of } j \text{ in } T\}.$$

Given two trees T_1 and T_2, the *ancestor-descendent distance* is defined as

$$\text{ADD}(T_1, T_2) \coloneqq |\, \phi_{AD}(T_1) \oplus \phi_{AD}(T_2)|.$$

Definition 3 (PD)**.** ([Gov+20]) The length of the unique (undirected) path between two vertices i and j in tree T is denoted by $\text{path}(i,j,T)$. Given two trees T_1 and T_2, the *path distance* is defined as

$$\text{PD}(T_1, T_2) \coloneqq \sum_{i<j} |\, \text{path}(i,j,T_1) - \text{path}(i,j,T_2)|.$$

Observation 2.1. For two mutation trees $T_1, T_2 \in \mathcal{T}_n$ we have

$$\text{PCD}(T_1, T_2) = \text{d}_{\textbf{adj},L_1}(T_1, T_2) = \text{d}^2_{\textbf{adj},L_2}(T_1, T_2),$$

$$\text{ADD}(T_1, T_2) = \text{d}_{\textbf{anc},L_1}(T_1, T_2) = \text{d}^2_{\textbf{anc},L_2}(T_1, T_2),$$
$$\text{PD}(T_1, T_2) = \text{d}_{\textbf{dist},L_1}(T_1, T_2).$$

Proof. Since the elements of the adjacency matrix are 0 or 1 we have

$$\text{d}^2_{\textbf{adj},L_2}(T_1, T_2) = L_2^2(\textbf{adj}(T_1) - \textbf{adj}(T_2)) = L_1(\textbf{adj}(T_1) - \textbf{adj}(T_2)) = \text{d}_{\textbf{adj},L_1}(T_1, T_2).$$

On the other hand,

$$\begin{aligned} L_2^2(\textbf{adj}(T_1) - \textbf{adj}(T_2)) &= \sum_i \sum_j |\textbf{adj}(T_1)[i,j] - \textbf{adj}(T_2)[i,j]|^2 \\ &= \sum_{(i,j)\in T_1,(i,j)\notin T_2} 1 + \sum_{(i,j)\notin T_1,(i,j)\in T_2} 1 \\ &= |\, \phi_{PC}(T_2) \oplus \phi_{PC}(T_2)| \\ &= \text{PCD}(T_1, T_2). \end{aligned}$$

Since the elements of the ancestry matrix are 0 or 1 we have

$$\text{d}^2_{\textbf{anc},L_2}(T_1, T_2) = L_2^2(\textbf{anc}(T_1) - \textbf{anc}(T_2)) = L_1(\textbf{anc}(T_1) - \textbf{anc}(T_2)) = \text{d}_{\textbf{anc},L_1}(T_1, T_2).$$

On the other hand,

$$\begin{aligned} L_2^2(\mathbf{anc}(T_1) - \mathbf{anc}(T_2)) &= \sum_i \sum_j |\mathbf{anc}(T_1)[i,j] - \mathbf{anc}(T_2)[i,j]|^2 \\ &= \sum_{\substack{i \text{ is ancestor of } j \text{ in } T_1 \\ i \text{ is not ancestor of } j \text{ in } T_2}} 1 + \sum_{\substack{i \text{ is not ancestor of } j \text{ in } T_1 \\ i \text{ is ancestor of } j \text{ in } T_2}} 1 \\ &= |\,\phi_{AD}(T_2) \oplus \phi_{AD}(T_2)| \\ &= \mathrm{ADD}(T_1, T_2). \end{aligned}$$

The proof for the path distance is the same. □

Corollary 1. Observation 2.1 and Proposition 1 imply that $\mathrm{d}^2_{\mathbf{adj},L_2}$ and $\mathrm{d}^2_{\mathbf{anc},L_2}$ are metrics over trees.

3 Consensus vs Centroid Tree

Lemma 1. Given a set of matrices $\mathcal{S} = \{\mathbf{A}_1, \ldots, \mathbf{A}_k\}$ whose entries are in $[0,1]$, a weight $\mathbf{w} = (w_1, \ldots, w_k)$ whose entries are also in $[0,1]$ with $\sum_{i=1}^k w_i \leq 1$, and a real number $r > 0$, the following cost functions are equal for any binary matrix $\mathbf{A} \in \{0,1\}^{n\times n}$,

1. $\mathrm{cost}_1(\mathbf{A}) \coloneqq L_1(\sum_{i=1}^k w_i \mathbf{A}_i - \mathbf{A})$,
2. $\mathrm{cost}_2(\mathbf{A}) \coloneqq \sum_{i=1}^k w_i L_1(\mathbf{A}_i - \mathbf{A})$.

Proof. Note that for any binary $\mathbf{A}$, we have

$$\mathrm{cost}_1(\mathbf{A}) = L_1(\sum_{i=1}^k w_i \mathbf{A}_i - \mathbf{A}) = \sum_{u,v} |\sum_{i=1}^k w_i \mathbf{A}_i[u,v] - \mathbf{A}[u,v]|.$$

Since $\mathbf{A}_i[u,v] \in [0,1]$ and $w_i \in [0,1]$ and $\sum_{i=1}^k w_i \leq 1$, we have

$$0 \leq \sum_{i=1}^k w_i \mathbf{A}_i[u,v] \leq 1,$$

and consequently,

$$\begin{aligned} \mathrm{cost}_1(\mathbf{A}) &= \sum_{u,v:\mathbf{A}[u,v]=0} \sum_{i=1}^k w_i \mathbf{A}_i[u,v] + \sum_{u,v:\mathbf{A}[u,v]=1} \left[1 - \sum_{i=1}^k w_i \mathbf{A}_i[u,v]\right] \\ &= L_1(\mathbf{A}) + \sum_{i=1}^k w_i \left[\sum_{u,v:\mathbf{A}[u,v]=0} \mathbf{A}_i[u,v] - \sum_{u,v:\mathbf{A}[u,v]=1} \mathbf{A}_i[u,v]\right]. \end{aligned}$$

For $\text{cost}_2(\mathbf{A})$ we have

$$\begin{aligned}
\text{cost}_2(\mathbf{A}) &= \sum_{i=1}^{k}\sum_{u,v} w_i|\mathbf{A}_i[u,v]-\mathbf{A}[u,v]| \\
&= \sum_{i=1}^{k}\left[\sum_{u,v:\mathbf{A}[u,v]=0} w_i\mathbf{A}_i[u,v] + \sum_{u,v:\mathbf{A}[u,v]=1} w_i(1-\mathbf{A}_i[u,v])\right] \\
&= \Sigma w_i \times \text{number of 1s in } \mathbf{A} + \sum_{i=1}^{k} w_i\left[\sum_{u,v:\mathbf{A}[u,v]=0}\mathbf{A}_i[u,v] - \sum_{u,v:\mathbf{A}[u,v]=1}\mathbf{A}_i[u,v]\right] \\
&= L_1(\mathbf{A}) + \sum_{i=1}^{k} w_i\left[\sum_{u,v:\mathbf{A}[u,v]=0}\mathbf{A}_i[u,v] - \sum_{u,v:\mathbf{A}[u,v]=1}\mathbf{A}_i[u,v]\right],
\end{aligned}$$

showing that, $\text{cost}_2(\mathbf{A}) = \text{cost}_1(\mathbf{A})$. □

Lemma 2. Given a set of real matrices $\mathcal{S} = \{\mathbf{A}_1, \ldots, \mathbf{A}_k\}$, a weight vector $\mathbf{w} = (w_1, \ldots, w_k)$ whose entries are also in $[0,1]$ for which $\sum_{i=1}^{k} w_i = 1$, for the following cost functions and an arbitrary matrix $\mathbf{A} \in \mathbb{R}^{n\times n}$

1. $\text{cost}_1(\mathbf{A}) \coloneqq L_2(\sum_{i=1}^{k} w_i\mathbf{A}_i - \mathbf{A})$,
2. $\text{cost}_2(\mathbf{A}) \coloneqq \sum_{i=1}^{k} w_i L_2^2(\mathbf{A}_i - \mathbf{A})$,

we have $\text{cost}_1(\mathbf{A}) = \text{cost}_2(\mathbf{A}) + \text{constant}$.

Proof. Since $\text{cost}_1(\mathbf{A})$ is always non-negative, one may consider optimizing $\text{cost}_1^2(\mathbf{A})$. Expanding $\text{cost}_1(\mathbf{A})$ we have

$$\begin{aligned}
\text{cost}_1^2(\mathbf{A}) &= L_2^2(\Sigma w_i\mathbf{A}_i - \mathbf{A}) \\
&= L_2^2(\Sigma w_i\mathbf{A}_i) + L_2^2(\mathbf{A}) - 2\langle \Sigma w_i\mathbf{A}_i, \mathbf{A}\rangle \\
&= \text{constant} + X(\mathbf{A}),
\end{aligned}$$

where,

$$X(\mathbf{A}) = L_2^2(\mathbf{A}) - 2\langle \Sigma w_i\mathbf{A}_i, \mathbf{A}\rangle.$$

For $\text{cost}_2(\mathbf{A})$ we have

$$\begin{aligned}
\text{cost}_2(\mathbf{A}) &= \sum w_i L_2^2(\mathbf{A}_i - \mathbf{A})^2 \\
&= \sum w_i(L_2^2(\mathbf{A}_i) + L_2^2(\mathbf{A}) - 2\langle \mathbf{A}_i, \mathbf{A}\rangle) \\
&= \Sigma w_i L_2^2(\mathbf{A}_i) + \Sigma w_i L_2^2(\mathbf{A}) - 2\langle \Sigma w_i\mathbf{A}_i, \mathbf{A}\rangle \\
&= \text{constant} + X(\mathbf{A}),
\end{aligned}$$

proving the claim. □

Theorem 1. Given a set of mutation trees $\mathcal{S} \subset \mathcal{T}_n$ and a normal weight function $w : \mathcal{S} \to \mathbb{R}_{\geq 0}$,

a) for any binary embedding $\varepsilon\colon \mathcal{T}_n \to \{0,1\}^{n\times n}$, the solution sets of $\mathrm{CeTP}_{\varepsilon,L1,w}$ and $\mathrm{CoTP}_{\mathrm{d}_{\varepsilon,L1},w}$ are identical.

b) for any embedding $\varepsilon\colon \mathcal{T}_n \to \mathbb{R}^{n\times n}$, the solution sets of $\mathrm{CeTP}_{\varepsilon,L_2,w}$ and $\mathrm{CoTP}_{\mathrm{d}^2_{\varepsilon,L_2},w}$ are identical.

Proof. Given a set of trees $\mathcal{S} = \{T_1, \ldots, T_k\}$, by setting $\mathbf{A_i} = \varepsilon(T_i)$ and $\mathcal{A} = \{\mathbf{adj}(T) \mid T \in \mathcal{T}_n\}$,

a) the proof is straightforward using Lemma 1 by minimizing costs over the set $\mathcal{A}$.

b) the proof is straightforward using Lemma 2 by minimizing costs over the set $\mathcal{A}$.

□

4 Nearest Mapped Tree

4.1 $\mathrm{NMTP}_{\mathbf{adj},L}$

Theorem 2. The problems $\mathrm{NMTP}_{\mathbf{adj},L_1}$ and $\mathrm{NMTP}_{\mathbf{adj},L_2}$ can be solved in $\mathcal{O}(n^3)$ time through finding a maximum weight spanning arborescence tree.

Proof.

a) Reduction for $\mathrm{NMTP}_{\mathbf{adj},L_2}$:

The *Chu–Liu/Edmonds algorithm* [CL65; Edm67] can be used to find a spanning arborescence of minimum (or maximum) weight. Its running time is of order $\mathcal{O}(VE)$ and since all weights can be nonzero, its worst case running time is of order $\mathcal{O}(n^3)$.

We treat $\mathbf{M}$ as the adjacency matrix of a weighted graph. Its maximum weight spanning arborescence $T_{\max}$ can be computed using Edmond's algorithm. Set $\mathbf{A}^* = \mathbf{adj}(T_{\max})$. We claim that $\mathbf{A}^*$ is the optimal matrix. Let T be a rooted tree on $\mathbb{Z}_n$ with $\mathbf{A}_T = \mathbf{adj}(T)$. For the L_2 norm we have

$$\begin{aligned} L_2(\mathbf{M} - \mathbf{A}_T)^2 &= \sum_{e_{ij}\in T} (m_{ij} - 1)^2 + \sum_{e_{ij}\notin T} m_{ij}^2 \\ &= \sum_{i,j} m_{ij}^2 + \sum_{e_{ij}\in T} 1 - 2m_{ij} \\ &= \|\mathbf{M}\|_F^2 + n - 1 - 2\sum_{e_{ij}\in T} m_{ij} \\ &= \text{constant} - 2\omega(T) = \text{cost}(T), \end{aligned}$$

where $\omega(T) = \sum_{e_{ij}\in T} m_{ij}$ is the weight of T. The tree $T_{\max}$ has the largest $\omega(T)$ among trees on $\mathbb{Z}_n$, hence it is a minimizer of $\text{cost}(T)$.

b) Reduction for $\text{NMTP}_{\mathbf{adj},L_1}$:

We treat $\mathbf{M}$ as the adjacency matrix of a weighted graph. Define three sets of edge indices as follows:

$$\begin{aligned} I_0 =& \{(i,j) \mid m_{ij} < 0\}, \\ I_{0,1} =& \{(i,j) \mid 0 \leq m_{ij} \leq 1\}, \\ I_1 =& \{(i,j) \mid m_{ij} > 1\}. \end{aligned}$$

Let T be a rooted tree on $\mathbb{Z}_n$ with $\mathbf{A}_T = \mathbf{adj}(T)$. For the L_1 norm we have

$$L_1(\mathbf{M} - \mathbf{A}_T) = \sum_{(i,j)\in I_0} a_{ij} - m_{ij} + \sum_{\substack{(i,j)\in I_{0,1} \\ e_{ij}\in T}} 1 - m_{ij} + \sum_{\substack{(i,j)\in I_{0,1} \\ e_{ij}\notin T}} m_{ij} + \sum_{(i,j)\in I_1} m_{ij} - a_{ij} =$$

$$\sum_{(i,j)\in I_0} -m_{ij} + \sum_{(i,j)\in I_1} m_{ij} + \sum_{\substack{(i,j)\in I_0 \\ e_{ij}\in T}} 1 + \sum_{\substack{(i,j)\in I_{0,1} \\ e_{ij}\in T}} 1 - \sum_{\substack{(i,j)\in I_1 \\ e_{ij}\in T}} 1 - \sum_{\substack{(i,j)\in I_{0,1} \\ e_{ij}\in T}} m_{ij} + \sum_{\substack{(i,j)\in I_{0,1} \\ e_{ij}\notin T}} m_{ij}.$$

By adding and subtracting $\sum_{\substack{(i,j)\in I_1 \\ e_{ij}\in T}} 1 + \sum_{\substack{(i,j)\in I_{0,1} \\ e_{ij}\in T}} m_{ij}$ we have

$$\begin{aligned} L_1(\mathbf{M} - \mathbf{A}_T) &= \sum_{(i,j)\in I_0} -m_{ij} + \sum_{(i,j)\in I_1} m_{ij} + \sum_{(i,j)\in I_{0,1}} m_{ij} + \sum_{e_{ij}\in T} 1 - 2\Big(\sum_{\substack{(i,j)\in I_1 \\ e_{ij}\in T}} 1 + \sum_{\substack{(i,j)\in I_{0,1} \\ e_{ij}\in T}} m_{ij}\Big) \\ &= \mathit{constant} - 2\Big(\sum_{\substack{(i,j)\in I_1 \\ e_{ij}\in T}} 1 + \sum_{\substack{(i,j)\in I_{0,1} \\ e_{ij}\in T}} m_{ij}\Big). \end{aligned}$$

This means that in order to minimize $L_1(\mathbf{M} - \mathbf{A}_T)$, the weight of edges in $I_{0,1}$ are important, while for I_0 and I_1, just the number of edges matter. According to the final formulation one may deduce that:

1. Incorporating edges having indices in I_0 to the tree neither increases nor decreases the cost.
2. Adding edges to the tree having indices in I_1 reduces the cost, with the highest possible cost of an edge being 1.
3. Adding edges to the tree having indices in $I_{0,1}$ reduces the cost by the amount equal to its weight.

Therefore, by trimming weights to the interval of $[0, 1]$ and finding the maximum weighted arborescence for this trimmed tree one obtains the optimal tree.

□

4.2 $\mathbf{NMTP}_{\mathbf{anc},L}$

Firstly, we provide a set of necessary and sufficient conditions for a matrix to be an ancestry matrix of a rooted tree.

Lemma 3. Matrix $\mathbf{X}$ is an ancestry matrix of a rooted tree *if and only if* all of the following constraints are satisfied:

1. The diagonal elements are zero.

2. There is a row with all entries equal to one, except the diagonal element.

3. For any two pairs of rows $\mathbf{x}_i$ and $\mathbf{x}_j$ one of the following conditions holds:

   - $\mathbf{x}_i.\mathbf{x}_j = 0$ `and` $\mathbf{X}[i,j] = \mathbf{X}[j,i] = 0$.
   - $\mathbb{1}(\mathbf{x}_i) \subsetneq \mathbb{1}(\mathbf{x}_j)$ `and` $\mathbf{X}[j,i] = 1$.
   - $\mathbb{1}(\mathbf{x}_j) \subsetneq \mathbb{1}(\mathbf{x}_i)$ `and` $\mathbf{X}[i,j] = 1$.

Proof. Given a matrix $\mathbf{X}$ satisfying Lemma 3's constraints, we construct a tree T with the ancestry matrix $\mathbf{X}$. First, we know that there is a row with all 1s but the diagonal element corresponding to the root r of tree T. Go through the following steps repeatedly:

1. Pick a row $\mathbf{x}_i$ with the largest number of ones (among vertices not yet picked for the tree T).

2. Find a vertex $\mathbf{x}_j$ in T having the least number of 1s for which $\mathbb{1}(\mathbf{x}_i) \subset \mathbb{1}(\mathbf{x}_j)$ and $\mathbf{X}[j,i] = 1$.

3. Set $\mathbf{x}_i$ as a new child for $\mathbf{x}_j$ in T.

We know that there exists such $\mathbf{x}_j$ since $\mathbb{1}(r)$ is a superset of ones for other rows and is the first vertex in the tree. We also know that $\mathbf{x}_j$ is unique since if we consider $\mathbf{x}_k$ and $\mathbf{x}_l$ such that $\mathbf{x}_i \subset \mathbf{x}_k$ and $\mathbf{x}_i \subset \mathbf{x}_l$ then $\mathbf{x}_k.\mathbf{x}_l \neq 0$. Hence, either $\mathbb{1}(\mathbf{x}_k) \subsetneq \mathbb{1}(\mathbf{x}_l)$ or $\mathbb{1}(\mathbf{x})_l \subsetneq \mathbb{1}(\mathbf{x})_k$, implying that one of them has more 1's. This way every row of $\mathbf{X}$ is placed as a vertex in T.

Now, we want to prove that $\mathbf{X}$ is the ancestry matrix of T. Firstly, note that by the transitivity of the $\subsetneq$ operator, if x_j is an ancestor of x_i, then $\mathbb{1}(\mathbf{x}_i) \subsetneq \mathbb{1}(\mathbf{x}_j)$. If $\mathbf{x}_k$ is the parent of $\mathbf{x}_i$ in T, by construction we know that $\mathbf{X}[k,i] = 1$. Since $\mathbf{x}_k$ is either $\mathbf{x}_j$ itself or a descendant of it, we have $\mathbb{1}(\mathbf{x}_k) \subset \mathbb{1}(\mathbf{x}_j)$, therefore $\mathbf{X}[j,i] = 1$. It remains to show that if $\mathbf{X}[j,i] = 1$, then $\mathbf{x}_j$ is an ancestor of $\mathbf{x}_i$ in T. We use induction on the depth of $\mathbf{x}_i$ in T. If the depth of $\mathbf{x}_j$ is 0, then it is the root vertex and there exist no j for which the condition is true, therefore the statement holds for the base case. Next, consider the case where the depth of $\mathbf{x}_i$ is $m+1$. Since $\mathbf{X}[j,i] = 1$ we know that $\mathbb{1}(\mathbf{x}_i) \subsetneq \mathbb{1}(\mathbf{x}_j)$. On the other hand, if $\mathbf{x}_k$ is the parent of $\mathbf{x}_j$, we have $\mathbf{x}_j.\mathbf{x}_k \neq 0$, since $\mathbf{X}[j,i] = \mathbf{X}[k,i] = 1$. But $\mathbf{x}_k$ is the row with the least number of 1's which is a superset of $\mathbf{x}_i$, implying that $\mathbb{1}(\mathbf{x}_k) \subsetneq \mathbb{1}(\mathbf{x}_j)$ and $\mathbf{X}[j,k] = 1$. Now, $\mathbf{x}_k$ is of depth m and by induction hypothesis we know that $\mathbf{x}_j$ is an ancestor of $\mathbf{x}_k$, hence the statement holds. This proves the *if* part. The *only if* part is clear by definitions. □

Auxiliary Theorem 1. Given a matrix $\mathbf{M} \in \mathbb{R}^{n \times n}$, the problem $\text{NMTP}_{\mathbf{anc},L_2}$ can be solved by AncL2 (Algorithm 1).

Algorithm 1: AncL2

$$
\begin{array}{llll}
\text{minimize} & \sum_{0\le i,j\le n} (x_{ij} - 2m_{ij}x_{ij}) & & \\
\text{subject to} & x_{ij} + x_{jk} - x_{ik} \le 1 & \forall i,j,k \in \mathbb{Z}_n & \text{(transitivity)} \\
& x_{ij} + x_{ji} \le 1 & \forall i,j \in \mathbb{Z}_n & \text{(no cycle)} \\
& x_{ik} + x_{jk} - x_{ij} - x_{ji} \le 1 & \forall i,j,k \in \mathbb{Z}_n, i \ne j \ne k \ne i & \text{(common descendant)} \\
& \sum_{j\ne i} x_{ij} = y_i & \forall i \in \mathbb{Z}_n & \text{(node out degree)} \\
& \sum_{0\le i\le n} z_i = 1 & & \text{(root index)} \\
& y_i \ge (n-1)z_i & \forall i \in \mathbb{Z}_n & \text{(there should be a root)} \\
& y_i \le (n-2)(1+z_i) & \forall i \in \mathbb{Z}_n & \text{(just one root)} \\
& x_{ij}, z_i \in \{0,1\} & \forall i,j \in \mathbb{Z}_n & \\
& 0 \le y_i \le n-1 & \forall i \in \mathbb{Z}_n &
\end{array}
$$

Proof. In all of the following lemmas the matrix $\mathbf{X}$ is the optimal solution for AncL2.

Note 4.1. The cost function of the AncL2 ILP is

$$\sum_{0\le i,j\le n} x_{ij} - 2m_{ij}x_{ij}, \tag{1}$$

but the raw cost obtained by direct application of L_2 norm is

$$\sum_{0\le i,j\le n} (m_{ij} - x_{ij})^2. \tag{2}$$

Since m_{ij}s are constant and x_{ij}s are 0 or 1, one may verify that

$$\sum_{0\le i,j\le n} (m_{ij} - x_{ij})^2 = \sum_{0\le i,j\le n} (m_{ij}^2 + x_{ij}^2 - 2m_{ij}x_{ij}) = \text{constant } + \sum_{0\le i,j\le n} x_{ij} - 2m_{ij}x_{ij},$$

therefore minimizing 2 and 1 are the same.

Note 4.2. Any tree in $\mathcal{T}_n$ would satisfy all of the constraints of AncL2 ILP, therefore the feasible region is not empty.

Lemma 4. The graph G having the adjacency matrix $\mathbf{X}$ is transitive.

Proof. If there is an edge connecting u to v and an edge connecting v to w, then we know that $x_{uv} = 1$ and $x_{vw} = 1$. Therefore, the "transitivity" condition $x_{uv} + x_{vw} - x_{uw} \le 1$ implies that $x_{uw} = 1$ and there exist an edge between u and w. □

Lemma 5. The graph G having the adjacency matrix $\mathbf{X}$ has no cycle.

Proof. First note that we have no self loop in G, since for each vertex i, using the "no cycle" condition $x_{ii} + x_{ii} \leq 1$, we have $x_{ii} = 0$. By Lemma 4 we know that G is transitive, therefore if we have a cycle of length greater than 1, for every pair of vertices u and v in the cycle we have $x_{uv} = 1$ and $x_{vu} = 1$ which contradicts the "no cycle" condition. □

Lemma 6. In the graph G having adjacency matrix $\mathbf{X}$, if vertices u and v are connected to the vertex k, i.e., $x_{uk} = x_{vk} = 1$, then either $x_{uv} = 1$ or $x_{vu} = 1$ holds, but not both.

Proof. This is a direct consequence of the "common descendant" condition and Lemma 5. □

Lemma 7. The matrix $\mathbf{X}$ which is a solution for AncL2 is an ancestry matrix.

Proof. For the proof we use Lemma 3.

1. Using Lemma 5 we know that all diagonal elements are zero.

2. Using the previous item and the "there should be a root" and the "just one root" conditions we know that there is a row in which all non-diagonal elements are equal to 1.

3. Considering two rows u and v,

   - if $x_{uv} = x_{vu} = 0$, then there is no w such that $x_{uw} = x_{vw} = 1$, since if there is such w, by Lemma 6 $x_{uv} = 1$ or $x_{vu} = 1$ which is a contradiction. Hence we have
   $$\sum_i x_{ui} x_{vi} = \mathbf{x}_u . \mathbf{x}_v = 0.$$
   - if $x_{uv} = 1$ and $x_{vu} = 0$ then by transitivity for each w where $x_{vw} = 1$, we have $x_{uw} = 1$, then
   $$\mathbb{1}(\mathbf{x}_v) \subsetneq \mathbb{1}(\mathbf{x}_u).$$
   - if $x_{uv} = 0$ and $x_{vu} = 1$ by a symmetrical argument we have
   $$\mathbb{1}(\mathbf{x}_u) \subsetneq \mathbb{1}(\mathbf{x}_v).$$
   - the case where $x_{uv} = 1$ and $x_{vu} = 1$ is not possible by Lemma 5.

□

By Note 4.1 and Lemma 7 we see that the optimal solution of AncL2 is the answer to the NMTP$_{\mathbf{anc},L_2}$ problem.

□

Note 4.3. By introducing a condition of the form $x_{ii} = 0$ for all i's, the two conditions "no cycle" and "just one root" may be omitted considering the "transivity" condition.

Auxiliary Theorem 2. Given a matrix $\mathbf{M} \in \mathbb{R}^{n \times n}$, the problem NMTP$_{\mathbf{anc},L_1}$ can be solved by AncL1 (Algorithm 2).

Proof. The proof is almost the same as the proof of Theorem 1. The cost function is different here and the variable u_{ij} is introduced to represent the value of $|x_{ij} - m_{ij}|$. □

Algorithm 2: AncL1

$$
\begin{array}{llll}
\text{minimize} & \sum_{0\le i,j\le n} u_{ij} & & \\
\text{subject to} & x_{ij} + x_{jk} - x_{ik} \le 1 & \forall i,j,k \in \mathbb{Z}_n & \text{(transitivity)} \\
& x_{ij} + x_{ji} \le 1 & \forall i,j \in \mathbb{Z}_n & \text{(no cycle)} \\
& x_{ik} + x_{jk} - x_{ij} - x_{ji} \le 1 & \forall i,j,k \in \mathbb{Z}_n, i \ne j \ne k \ne i & \text{(common descendant)} \\
& \sum_{j\ne i} x_{ij} = y_i & \forall i \in \mathbb{Z}_n & \text{(node out degree)} \\
& \sum_{0\le i\le n} z_i = 1 & & \text{(root index)} \\
& y_i \ge (n-1)z_i & \forall i \in \mathbb{Z}_n & \text{(there should be a root)} \\
& y_i \le (n-2)(1+z_i) & \forall i \in \mathbb{Z}_n & \text{(just one root)} \\
& x_{ij}, z_i \in \{0,1\} & \forall i,j \in \mathbb{Z}_n & \\
& 0 \le y_i \le n-1 & \forall i \in \mathbb{Z}_n & \\
& u_{ij} \in \mathbb{R} & \forall i,j \in \mathbb{Z}_n & \\
& x_{ij} - m_{ij} \le u_{ij} & \forall i,j \in \mathbb{Z}_n & \\
& m_{ij} - x_{ij} \le u_{ij} & \forall i,j \in \mathbb{Z}_n &
\end{array}
$$

4.3 $\text{NMTP}_{\mathbf{dist},L}$

4.3.1 $\mathcal{NP}$-hardness

Definition 4. Partition Into Triangles (**PIT**) : *Given a graph $G = (V, E)$ where $|V| = 3k$ for some $k \in \mathbb{N}$ and each $v \in V$ is covered by some triangles in G, can V be partitioned into disjoint sets $V_1, \ldots, V_k$ each containing exactly 3 vertices, such that each of these V_is is the vertex set of a triangle in G?*

Auxiliary Theorem 3. ([GJ79]) The problem Partition Into Triangles is $\mathcal{NP}$-complete.

Note 4.4. The original problem stated in [GJ79] does not have the condition that each vertex should be covered by some triangle in the given graph G. But adding this condition does not change the complexity since it is possible to be checked in polynomial time.

Theorem 3. The problems $\text{NMTP}_{\mathbf{dist},L_1}$ and $\text{NMTP}_{\mathbf{dist},L_2}$ are $\mathcal{NP}$-hard.

Proof. Given a graph $G = (V, E)$ with $|V| = n = 3z$ and p triangles, we want to reduce PIT to $\text{NMTP}_{\mathbf{dist},L_1}$ and $\text{NMTP}_{\mathbf{dist},L_2}$. We have to create a matrix $\mathbf{M}$ based on G and solve $\text{NMTP}_{\mathbf{dist},L}$ ($L \in \{L_1, L_2\}$) for $\mathbf{M}$ and create a triangulation of V if there exist one using its solution. Since optimizing with respect to L_2 and L_2^2 are exactly the same, for simplicity of calculations, we consider L_2^2 in what follows.

In order to create $\mathbf{M}$, consider a graph G' with vertices and edges in 5 layers described as,

1. Layer-R: A single vertex r.

2. Layer-S: A set of n^5 vertices. Each vertex in this layer is denoted by s_i. All of them are connected directly to r.

3. Layer-S2: The union of n^5 sets of n^5 vertices: For each s_i in Layer-S there are n^5 vertices denoted by s_{ij} directly connected to it.

4. Layer-TR: A set of p vertices for each triangle tr_i in G. Each vertex denoted by t_i, is directly connected to r.

5. Layer-V: The set V. Each vertex v in this layer is connected to every t_i for which $v \in \text{tr}_i$.

Observation 4.1. The distances between different layers of vertices in G' are described in the following table.

	r	s_j	s_{op}	t_y	u
s_i	1	0 if $i = j$<br>2 if $i \neq j$			
s_{kl}	2	1 if $j = k$<br>3 if $j \neq k$	0 if $o = k$ and $l = p$<br>2 if $o = k$ and $l \neq p$<br>4 if $o \neq k$		
t_x	1	2	3	0 if $x = y$<br>2 if $x \neq y$	
v	2	3	4	1 if $v \in \text{tr}_y$<br>3 if $v \notin \text{tr}_y$	0 if $u = v$<br>2 if $\exists \text{tr}_h : u, v \in \text{tr}_h$<br>4 if $\nexists \text{tr}_h : u, v \in \text{tr}_h$

Now define the matrix $\mathbf{M}$ whose entries are identical to those of $\mathbf{dist}(G')$ except for all (pairs of) vertices of Layer-V for which we define the entries as

$$\mathbf{M}[u, v] = \begin{cases} 0 & \text{if } u = v, \\ 3 - \epsilon & \text{if } \exists \text{tr}_h : u, v \in \text{tr}_h \text{ and } u \neq v, \\ 3 & \text{if } \nexists \text{tr}_h : u, v \in \text{tr}_h \text{ and } u \neq v, \end{cases}$$

where ϵ is a small nonzero real number to be set later. Let tree T with distance matrix $\mathbf{D}$ be an optimal solution of $\text{NMTP}_{\mathbf{dist},L}$ for $\mathbf{M}$. It is obvious that $V(T) = V(G')$. We first want to show that $E(G') - E(T) \subset$ Layer-V $\times$ Layer-TR.

Observation 4.2. *We see that $L(\mathbf{M} - \mathbf{D}) = \mathcal{O}(n^4)$ for $L \in \{L_1, L_2^2\}$.* Consider a tree T' with all edges between vertices in layers Layer-R, Layer-S, Layer-S2, and Layer-TR from G'. Then we connect any v in Layer-V to exactly one t_i in Layer-TR. Set $\mathbf{D}' = \mathbf{dist}(T')$. In T' the distance between vertices in three layers Layer-R, Layer-S, and Layer-S2 to all vertices in G' are the same as in $\mathbf{M}$. Furthermore the distance between vertices in Layer-TR with each other, is also consistent with $\mathbf{M}$. The only distances that may not match with $\mathbf{M}$ are distances between vertices in Layer-V and vertices in Layer-V or Layer-TR. Because there are p triangles in G, assuming $0 < \epsilon < 1$ we have $L(\mathbf{M} - \mathbf{D}') \leq 2pn + (1 + \epsilon)^2 \binom{n}{2}$. Since $p = \mathcal{O}(n^3)$, we have $L(\mathbf{M} - \mathbf{D}) \leq L(\mathbf{M} - \mathbf{D}') = \mathcal{O}(n^4)$.

Observation 4.3. *In the tree T, the vertex r remains directly connected to all vertices in Layer-S.* By Observation 4.2, r remains directly connected to $\Omega(n^5)$ vertices from Layer-S. Now suppose that there exist a vertex s_i from Layer-S, for which $(r, s_i) \notin E(T)$. Since T is a tree, there is just one path between r and s_i. If we remove one edge in this path, s_i becomes disconnected from r. Since we just removed one vertex, r is still connected directly to $\Omega(n^5)$ vertices from Layer-S. This means the distance between s_i and $\Omega(n^5)$ vertices from Layer-S is at least 3, which makes $L(\mathbf{M} - \mathbf{D}) = \Omega(n^5)$ which is a contradiction.

Observation 4.4. *In the tree T, all vertices in Layer-$S2$ remain directly connected to the corresponding vertices from Layer-S.* The proof is similar to Observation 4.3.

Observation 4.5. *In the tree T, all vertices in Layer-TR remain directly connected to r.* Vertex r is directly connected to all vertices in Layer-S. A similar argument to Observation 4.3 reveals that the distance for all vertices in Layer-TR to r should remain 1.

Observation 4.6. *In the tree T, all vertices in Layer-V remain directly connected to vertices in Layer-TR.* Consider a vertex v from Layer-V. If it is connected directly to a vertex from Layer-S2 like s_{ij}, then its distance to $\Omega(n^5)$ vertices connected to s_i reduces from 4 to 3. If it is directly connected to a vertex from Layer-S like s_i, then its distance to $\Omega(n^5)$ vertices connected to s_i reduces from 4 to 2. If it is directly connected to the single vertex in Layer-R namely r, then its distance to $\Omega(n^5)$ vertices connected to r reduces from 3 to 2. Thus v cannot be connected directly to any vertex in any of the Layer-R, Layer-S or Layer-S2. It also cannot be connected directly to any other vertex u from Layer-V, because it makes their distance unequal to all other vertices in the tree, because for any vertex in T, it is either closer to v or u. Considering just vertices in Layer-S2, if v is directly connected to u, then $L(\mathbf{M} - \mathbf{D}) = \Omega(n^{10})$. Therefore, vertices in Layer-V are directly connected to vertices in Layer-TR.

Observation 4.7. *In the tree T, each vertex v from Layer-V is directly connected to one vertex in Layer-TR.* Since all vertices in Layer-TR are directly connected to r, v cannot be connected to two vertices in Layer-TR.

Now we want to show that if there exist a triangulation of G, the vertices from Layer-V, are arranged in T in a way that this triangulation can be easily constructed.

Observation 4.8. *In the tree T, each v from Layer-V is connected to a t_i from Layer-TR for which $v \in tr_i$.* Suppose $v \notin \mathrm{tr}_i$, then let T' be a tree obtained by disconnecting v from t_i and connect it to some t_j for which $v \in \mathrm{tr}_j$. Defince $X(x) = \{u \in V \mid u \text{ is connected to } t_x\}$. Then the distances of T and T' are identical except for distances between v and vertices in $(\{t_i, t_j\} \cup X(i) \cup X(j)) - \{v\}$. The difference of sum of the distances between v and

$X(i) \cup X(j)$ in T and T' for $L \in \{L_1, L_2^2\}$ is

$$\begin{aligned} c = & \sum_{u \in X(i) \cup X(j)} L(\mathbf{M}[u,v] - \mathbf{dist}(T')[u,v]) - L(\mathbf{M}[u,v] - \mathbf{dist}(T)[u,v]) \\ \leq & \sum_{u \in V} L(\mathbf{M}[u,v] - \mathbf{dist}(T')[u,v]) - L(\mathbf{M}[u,v] - \mathbf{dist}(T)[u,v]) \\ \leq & \sum_{u \text{ is co-triangle with } v} L(\mathbf{M}[u,v] - \mathbf{dist}(T')[u,v]) - L(\mathbf{M}[u,v] - \mathbf{dist}(T)[u,v]) \\ + & \sum_{u \text{ is not co-triangle with } v} L(\mathbf{M}[u,v] - \mathbf{dist}(T')[u,v]) - L(\mathbf{M}[u,v] - \mathbf{dist}(T)[u,v]) \\ \leq & \sum_{u \text{ is co-triangle with } v} L(\mathbf{M}[u,v] - \mathbf{dist}(T')[u,v]) - L(\mathbf{M}[u,v] - \mathbf{dist}(T)[u,v]), \end{aligned}$$

since if u is not co-triangle with v we have

$$L(\mathbf{M}[u,v] - \mathbf{dist}(T')[u,v]) = (\mathbf{M}[u,v] - \mathbf{dist}(T)[u,v]) = 1.$$

Now, for L_1 we have

$$c \leq \sum_{u \text{ is co-triangle with } v} 1 + \epsilon - (1 - \epsilon) = 2k\epsilon,$$

and for L_2^2 we have

$$c \leq \sum_{u \text{ is co-triangle with } v} (1 + \epsilon)^2 - (1 - \epsilon)^2 = 4k\epsilon,$$

where k is the number of vertices which are co-triangle with v. As a result, if we choose ϵ small enough, c is bounded by 1. Hence, the distance between v and t_i is increased from 1 to 3 and distance between v and t_j is decreased from 3 to 1. This means that $\mathbf{dist}(T')$ is closer to the $\mathbf{M}$ from $\mathbf{D}$, which is a contradiction.

Observation 4.9. *If every vertex v from Layer-V in T has exactly two other siblings, then this gives a triangulation of G.* By Observations 4.7 and 4.8, if one considers the sets $V_i = \{v | v \in \text{Layer-V and } (v, t_i) \in E(T)\}$, there are $z = \frac{n}{3}$ nonempty V_i which are all disjoint and each of them having exactly 3 members and covering corresponding triangle tr_i.

Observation 4.10. *If there is a triangulation of V, then T will specify one.* If for every v in Layer-V, it has two siblings from Layer-V connected to the same t_i, then, T gives us a triangulation. If T is not giving a triangulation, suppose T' is a tree obtained by one of the triangulations of G. By Observation 4.8 we know that in T every vertex v from Layer-V has at most two siblings. The distances between all vertices in all layers except Layer-V are the same for both T and T'. The distances between Layer-V and three layers Layer-R, Layer-S and Layer-S2 is the same too for both trees. The distances between Layer-V and Layer-TR may be different for some vertices. But the difference between distances sums up to zero,

because every vertex is connected to exactly one of it's covering triangles. It has distance 1 to it and distance 3 to all other triangles.

Now, consider the difference between distances of vertices in Layer-V in the input matrix $\mathbf{M}$. For a vertex v in T with q siblings ($0 \le q \le 2$), in the case of L_1 norm we have

$$\begin{aligned}\sum_{u \in V} |\mathbf{M}[u,v] - \mathbf{D}[u,v]| &= q((3-\epsilon)-2) + (k-q)(4-(3-\epsilon)) + (n-k-1)(4-3)\\ &= q - q\epsilon + k - q + (k-q)\epsilon + n - k - 1\\ &= n - 1 + (k-2q)\epsilon.\end{aligned}$$

For T' we have

$$\sum_{u \in V} |\mathbf{M}[u,v] - \mathbf{dist}(T')[u,v]| = n - 1 + (k-4)\epsilon.$$

For a vertex v in T with q siblings ($0 \le q \le 2$), in the case of L_2^2 norm we have

$$\begin{aligned}\sum_{u \in V} (\mathbf{M}[u,v] - \mathbf{D}[u,v])^2 &= q((3-\epsilon)-2)^2 + (k-q)(4-(3-\epsilon))^2 + (n-k-1)(4-3)^2\\ &= q + q\epsilon^2 - 2q\epsilon + k - q + (k-q)\epsilon^2 + 2(k-q)\epsilon + n - k - 1\\ &= n - 1 + k\epsilon^2 + 2(k-2q)\epsilon.\end{aligned}$$

For T' and norm L_2^2 we have

$$\sum_{u \in V} (\mathbf{M}[u,v] - \mathbf{dist}(T')[u,v])^2 = n - 1 + k\epsilon^2 + 2(k-4)\epsilon.$$

The tree T does not impose a triangulation, therefore there exist a v in Layer-V with $q < 2$, accordingly for $L \in \{L_1, L_2^2\}$ we have $L(\mathbf{M} - \mathbf{dist}(T')) < L(\mathbf{M} - \mathbf{D})$ which is a contradiction.

We used the parameter ϵ in Observations 4.2, 4.8, and 4.10 and it is straight forward to verify that If one chooses

$$\epsilon = \frac{1}{4n},$$

then all observations are true.

□

4.3.2 ILPs

Auxiliary Theorem 4. Given a matrix $\mathbf{M} \in \mathbb{R}^{n \times n}$, the problem $\text{NMTP}_{\mathbf{dist},L_1}$ can be solved by DistL1 (Algorithm 3).

Proof. The solution to the DistL1 is a graph G with the adjacency matrix E for which $E[i,j] = e_{ij}$. First, note that E is symmetric, irreflexive and has $n-1$ undirected edges. Thus, in order to prove that it is a tree with minimum distance from $\mathbf{M}$, it suffices to show that it has no cycles and also $\mathbf{D}$ is its distance matrix.

Algorithm 3: DistL1

$$
\begin{array}{llll}
\text{minimize} & \displaystyle\sum_{0\le i,j\le n} |d_{ij} - m_{ij}| & & \\
\text{subject to} & d_{ij} - d_{jk} - d_{ik} \le 0 & \forall i,j,k \in \mathbb{Z}_n & \text{(triangle equality)} \\
& d_{ij} - d_{ji} = 0 & \forall i,j \in \mathbb{Z}_n & \text{(symmetric)} \\
& d_{ii} = 0 & \forall i \in \mathbb{Z}_n & \text{reflexive} \\
& e_{ij} = e_{ji} & \forall i,j \in \mathbb{Z}_n & \text{(undirected)} \\
& e_{ii} = 0 & \forall i \in \mathbb{Z}_n & \text{(no self loop)} \\
& \displaystyle\sum_{j\ne i} e_{ij} = 2(n-1) & & \text{(number of edges)} \\
& e_{ij} + d_{ij} \ge 2 & \forall i,j \in \mathbb{Z}_n, i \ne j & \\
& \displaystyle\sum_{0\le k\le n} z_{ijk} = d_{ij} - 1 & \forall i,j \in \mathbb{Z}_n, i \ne j & \text{(vertices between } i \text{ and } j\text{)} \\
& z_{ijk} + z_{ikj} + z_{jki} \le 1 & \forall i,j,k \in \mathbb{Z}_n & \\
& z_{ijk} - z_{jik} = 0 & \forall i,j,k \in \mathbb{Z}_n & \\
& z_{iik}, z_{ikk} = 0 & \forall i,k \in \mathbb{Z}_n & \\
& e_{ij} - z_{ikj} - z_{jki} \le 0 & \forall i,j,k \in \mathbb{Z}_n, k \ne i,j & \\
& d_{ij} + 2n(1 - z_{ijk}) \ge d_{ik} + d_{kj} & \forall i,j,k \in \mathbb{Z}_n, k \ne i,j & \text{(additive distance)} \\
& d_{ij} \in \{1, \dots n-1\} & \forall i,j \in \mathbb{Z}_n, i \ne j & \\
& e_{ij}, z_{ijk} \in \{0,1\} & \forall i,j,k \in \mathbb{Z}_n &
\end{array}
$$

Note 4.5. Any tree in $\mathcal{T}_n$ would satisfy all of the constraints of AncL2 ILP, therefore the feasible region is not empty.

Observation 4.11. *Variables* $e_{ij} = 1$ *iff* $d_{ij} = 1$. If $d_{ij} = 1$ then by condition $e_{ij} + d_{ij} \ge 2$ we have that $e_{ij} = 1$. If $d_{ij} > 1$ then $\sum_k z_{ijk} > 0$ and thus $K = \{k \mid z_{ijk} = 1\}$ is not empty. Suppose $z_{ijk} = 1$ then by condition $z_{ijk} + z_{ikj} + z_{jki} \le 1$ and as a result $z_{ikj} = z_{jki} = 0$. Now by condition $e_{ij} - z_{ikj} - z_{jki} \le 0$ we know that $e_{ij} = 0$.

Observation 4.12. *The graph* G *is connected and every pair of vertices* u *and* v *are connected by a path of length* d_{uv}. We know that $d_{uv} \ge 1$. Let us use strong induction for the proof. For the base case, if $d_{uv} = 1$ then by Observation 4.11 we know that there is an edge between them, therefore they are connected with a path of length 1. Now assume that for $d = 1$ to m the statement holds. If $d_{uv} = m+1$ then by condition "vertices between i and j" there exist k for which $z_{uvk} = 1$. By "triangle equality" condition we know $d_{uv} \le d_{uk} + d_{kv}$ and by "additive distance" condition we know $d_{uv} \ge d_{uk} + d_{kv}$, therefore $d_{uv} = d_{uk} + d_{kv}$. Since $d_{uk} \le m$ and $d_{kv} \le m$, by induction hypothesis there exist a path of length d_{uk} from u to k and a path of length d_{kv} from k to v. Hence, u and v are connected by a path of length $d_{uv} = d_{uk} + d_{kv}$ passing through k.

This proves that G is indeed a tree, since it has $n-1$ edges and is connected. Also we proved that $\mathbf{dist}(G)[u,v] = d_{uv}$. Any tree satisfies all of these conditions, therefore the tree obtained by solving this ILP is a solution to $\text{NMTP}_{\mathbf{dist},L_1}$.

□

Auxiliary Theorem 5. Given a matrix $\mathbf{M} \in \mathbb{R}^{n\times n}$, the problem $\text{NMTP}_{\mathbf{dist},L_2}$ can be solved by DistL2 (Algorithm 4).

Algorithm 4: DistL2

$$
\begin{array}{llll}
\text{minimize} & \sum_{0\le i,j\le n} (d_{ij} - m_{ij})^2 & & \\
\text{subject to} & d_{ij} - d_{jk} - d_{ik} \le 0 & \forall i,j,k \in \mathbb{Z}_n & \text{(triangle equality)} \\
 & d_{ij} - d_{ji} = 0 & \forall i,j \in \mathbb{Z}_n & \text{(symmetric)} \\
 & d_{ii} = 0 & \forall i \in \mathbb{Z}_n & \text{reflexive} \\
 & e_{ij} = e_{ji} & \forall i,j \in \mathbb{Z}_n & \text{(undirected)} \\
 & e_{ii} = 0 & \forall i \in \mathbb{Z}_n & \text{(no self loop)} \\
 & \sum_{j\ne i} e_{ij} = 2(n-1) & & \text{(number of edges)} \\
 & e_{ij} + d_{ij} \ge 2 & \forall i,j \in \mathbb{Z}_n, i\ne j & \\
 & \sum_{0\le k\le n} z_{ijk} = d_{ij} - 1 & \forall i,j \in \mathbb{Z}_n, i\ne j & \text{(vertices between } i \text{ and } j\text{)} \\
 & z_{ijk} + z_{ikj} + z_{jki} \le 1 & \forall i,j,k \in \mathbb{Z}_n & \\
 & z_{ijk} - z_{jik} = 0 & \forall i,j,k \in \mathbb{Z}_n & \\
 & z_{iik}, z_{ikk} = 0 & \forall i,k \in \mathbb{Z}_n & \\
 & e_{ij} - z_{ikj} - z_{jki} \le 0 & \forall i,j,k \in \mathbb{Z}_n, k\ne i,j & \\
 & d_{ij} + 2n(1 - z_{ijk}) \ge d_{ik} + d_{kj} & \forall i,j,k \in \mathbb{Z}_n, k\ne i,j & \text{(additive distance)} \\
 & d_{ij} \in \{1,\dots n-1\} & \forall i,j \in \mathbb{Z}_n, i\ne j & \\
 & e_{ij}, z_{ijk} \in \{0,1\} & \forall i,j,k \in \mathbb{Z}_n &
\end{array}
$$

Auxiliary Theorem 6. Given a matrix $\mathbf{M} \in \mathbb{R}^{n\times n}$, the problem $\text{NMTP}_{\mathbf{dist},L_\infty}$ can be solved by DistLInf (Algorithm 5).

Proof. The variable u here is an upper bound for $L_1(M-X)$. Other parts of the proof is the same as Theorem 4. □

5 Implementation Details

Algorithms and simulations are implemented on Python (3.7.9). ILPs are solved by IBM ILOG CPLEX Optimizer (12.9) with DOcplex (2.23.222) python API. The algorithms are executed on an HPC with 20 cores and 8GB RAM dedicated to each instance of the problem, with a time limit of 4 hours per job.

ILPs for **dist** embedding have more variables and conditions than ILPs for **anc**, and consequently, DistL1 and DistLInf are not solved for $n = 20$ within 4 hours. The quadratic programming DistL2 cannot be solved in the time limit even for $n = 10$, so it was omitted from the analyses.

Algorithm 5: DistLInf

$$
\begin{array}{llll}
\text{minimize} & u & & \\
\text{subject to} & d_{ij} - d_{jk} - d_{ik} \leq 0 & \forall i, j, k \in \mathbb{Z}_n & \text{(triangle equality)} \\
& d_{ij} - d_{ji} = 0 & \forall i, j \in \mathbb{Z}_n & \text{(symmetric)} \\
& d_{ii} = 0 & \forall i \in \mathbb{Z}_n & \text{(reflexive)} \\
& e_{ij} = e_{ji} & \forall i, j \in \mathbb{Z}_n & \text{(undirected)} \\
& e_{ii} = 0 & \forall i \in \mathbb{Z}_n & \text{(no self loop)} \\
& \sum_{j \neq i} e_{ij} = 2(n-1) & & \text{number of edges} \\
& e_{ij} + d_{ij} \geq 2 & \forall i, j \in \mathbb{Z}_n, i \neq j & \\
& \sum_{0 \leq k \leq n} z_{ijk} = d_{ij} - 1 & \forall i, j, k \in \mathbb{Z}_n, i \neq j & \text{(vertices between } i \text{ and } j) \\
& z_{ijk} + z_{ikj} + z_{jki} \leq 1 & \forall i, j, k \in \mathbb{Z}_n & \\
& z_{ijk} - z_{jik} = 0 & \forall i, j, k \in \mathbb{Z}_n & \\
& z_{iik} = 0 & \forall i, k \in \mathbb{Z}_n & \\
& e_{ij} - z_{ikj} - z_{jki} \leq 0 & \forall i, j, k \in \mathbb{Z}_n, k \neq i, j & \\
& d_{ij} + 2n(1 - z_{ijk}) \geq d_{ik} + d_{kj} & \forall i, j, k \in \mathbb{Z}_n, k \neq i, j & \text{(additive distance)} \\
& d_{ij} \in \{1, \ldots n-1\} & \forall i, j \in \mathbb{Z}_n, i \neq j & \\
& e_{ij}, z_{ijk} \in \{0, 1\} & \forall i, j, k \in \mathbb{Z}_n & \\
& d_{ij} - m_{ij} \leq u & \forall i, j \in \mathbb{Z}_n & \\
& m_{ij} - d_{ij} \leq u & \forall i, j \in \mathbb{Z}_n & \\
& u \in \mathbb{R} & &
\end{array}
$$

6 Simulation Method

The function `random_tree` from the NetworkX(2.6.3) package, with default parameters, is used to generate random trees. For reproducibility, a seed parameter is used for every random function involved in generating and altering trees. The generation of CCFs (Cancer Cell Fraction) is done in a top-down manner. The CCF for root vertex is 1. To generate the CCFs for k children of a vertex v with known CCF, we generate $k+1$ uniform random numbers in $[0, 1]$ and normalize them so that their sum is equal to CCF(v) and assign first k normalized values to the children. Three types of possible alterations can be applied to the generated ground truth trees:

1. **pc** (parent-child): This involves swapping a child vertex with its parent. Th substitution is perforemed if

   $$r\text{CCF}(parent) > \text{CCF}(parent) - \text{CCF}(child).$$

   Where r is a random number in $[0, 1]$. The child vertex is uniformly picked from the vertices of the tree. We also swap the CCFs for the parent and child vertices so that CCF values remain consistent with the new structure of the tree.

2. **bm** (branch-move): This involves moving a vertex with all of its descendants to a vertex which has enough *empty CCF*. The empty CCF of vertex v is defined as

$$\mathrm{CCF}(v) - \sum_{u \text{ is a child of } v} \mathrm{CCF}(u).$$

The vertex v is uniformly selected from tree vertices. Then possible parents, i.e., vertices with enough empty CCF are computed. If the set of possible parents is not empty, one of them is selected uniformly as the new parent.

3. **nr** (node-remove): This involves removing a vertex from its place in the tree and placing it as a direct child of the root vertex. Its former parent becomes the new parent of its former children. Firstly, vertex v is selected uniformly from the tree vertices. Then it will become a direct child of the root vertex if

$$r > \mathrm{CCF}(v).$$

where r is a random number in $[0, 1]$. After moving the vertex v, we set $\mathrm{CCF}(v) = \epsilon$ because the root vertex may not have any empty CCF. We used $\epsilon = 10^{-6}$.

A parameter **cp** (change-probability) is used to determine the number of alterations applied to each tree. For each alteration, we repeatedly generate a random number $r \in [0, 1]$. If $r >$ **cp** we apply the alteration. If not, we stop applying this type of alteration and move on to the next type of alteration. The order of application of alterations is the same as listed above. The source code is also available at the git repository associated with this paper.

7 Ranking Table

Table 1: Best performing algorithms in each simulation setting. First and second algorithms with smallest average CASet distance (the number in the parenthesis) to the ground truth trees are reported for different simulation settings.

**pc**, **bm**, **nr***	$\frac{k}{n} = \frac{1}{2}$	$\frac{k}{n} = 1$	$\frac{k}{n} = 2$
$1, 1, 0$	1-GraPhyC(0.06)<br>1-2-**AncL2**(0.06)<br>2-ConTreeDP(0.13)	1-GraPhyC(0.02)<br>1-2-**AncL2**(0.04)<br>2-ConTreeDP(0.11)	1-GraPhyC(0.00)<br>1-2-**AncL2**(0.02)<br>2-ConTreeDP(0.07)
$1, 0, 0$	1-ConTreeDP(0.00)<br>2-GraPhyC(0.01)<br>2-TuELiP(0.01)<br>2-2-**AncL2**(0.02)	1-GraPhyC(0.00)<br>1-TuELiP(0.00)<br>1-ConTreeDP(0.00)<br>1-2-**AncL2**(0.00)	1-GraPhyc(0.00)<br>1-TuELiP(0.00)<br>1-2-**AncL2**(0.00)<br>1-ConTreeDP(0.00)
$0, 1, 0$	1-GraPhyC(0.00)<br>2-2-**AncL2**(0.04)	1-GraPhyC(0.00)<br>2-2-**AncL2**(0.03)	1-GraPhyC(0.00)<br>2-2-**AncL2**(0.02)
0 or $1, 0$ or $1, 1$	1-3-**AncL2**(0.12)<br>2-2-**AncL2**(0.16)	1-3-**AncL2**(0.07)<br>2-2-**AncL2**(0.15)	1-3-**AncL2**(0.04)<br>2-2-**AncL2**(0.13)

* Parent-child, branch-move, and node-remove parameters for simulation method. The numbers 1 and 0 represent *including* or *excluding* the corresponding alteration, respectively.

- The significance threshold for p-values is 10^{-3}. Given ADD distances of the outputs of algorithms to the ground truth tree, for every pair of algorithms, the p-value is calculated using paired t-test. When the normality condition is not satisfied we have used Kolmogorov-Smirnov test. The null hypothesis is: "distances have the same mean".
- When there is no statistical evidence for the superiority of one method, all of the best methods are reported. The number before the name of an algorithm, shows the rank of the algorithm based on its performance. All algorithms in a cell are sorted by the mean value of distances to the ground truth tree.
- Algorithms in bold are introduced in this article.
- The change probability (**cp**) parameter is set to be equal to 0.9.
- For the last row, the average CASet distance is reported for the case where $\mathbf{pc} = 1, \mathbf{bm} = 1, \mathbf{nr} = 1$.

8 Single-labeled AncL2 vs Multi-labeled TuELiP

Even in cases where the ground truth tree is multi-labeled, it is still justifiable to look for the nearest single-labeled tree to this multi-labeled ground truth solution as an approximation. This approach of approximating a multi-labeled solution by a single-labeled one is in coherence with our setup of looking for a nearest neighbour tree formalized as the NMTP problem. On the other hand, confining the search space to single-labeled trees may be considered as a regularization process for the corresponding optimization problems that definitely gives rise to simpler and more efficient algorithms. Hence, from this point of view and considering the fact that usually all existing methods output an approximation of the exact ground truth solution, one may consider the single-labeled case as an important case to be studied even if one is dealing with multi-labeled data. In what follows, we have done our best to provide some evidence that in our approach this is actually the case.

In order to provide some evidence on validity of approximating multi-labeled trees by single-labeled ones, in addition to what have already appeared in the original version of the article regarding comparing all algorithms on our single-labeled simulated dataset, we have designed the following comparison setups on multi-labeled datasets to show that our single-labeled algorithm may be considered as a reliable algorithm compared to the existing methods available.

To mention the main subtlety here, note that in order to compare the performance of TuELiP on a multi-labeled data to the performance of its single-labeled counterpart AncL2, one must be careful about the fact that our definition of the ancestry matrix is just applicable to single-labeled trees. Considering the definition of *ancestry* relations defined in the TuELiP paper (Paragraph 2, Section 2.2 in [GSO23]), one may generate the ancestry matrices of multi-labeled input trees and find their centroid matrix (i.e. say the matrix $\mathbf{A}$). Now, applying our new results on the NMTP setup, one may use AncL2 to find a single-labeled tree T as the best single-labeled approximation ancestry tree for $\mathbf{A}$. This centroid tree T may be considered as an approximation for the solution of TuELiP. Clearly, this experiment setup provides a fair information about the performance of our single-labeled method when it is applied to a multi-labeled dataset.

We have elaborated on applying the above mentioned scenario to a couple of relevant cases as follows (for the algorithms ConTreeDP and TuELiP):

- **ConTreeDP dataset:** The synthetic data used in ConTreeDP paper is published and is available online at its GitHub page. We have used this dataset to computed the CASet distance between single-labeled outputs of AncL2 and the ground truth trees as well as the CASet distance between multi-labeled outputs of TuELiP (i.e. the most up-to-date consensus method available) to the ground truth trees. As depicted in Table 2, in 5 out of 9 cases the two algorithms have equal average distance to the ground truth up to 3 decimal points and each of them have better outputs in 2 cases, while none of the p-values for paired t-tests are significant. Hence, we have not been able to trace any significant evidence on the advantage of multi-labeled setup compared to the single-labeled one, regardless of the cases when ground truth trees are in fact multi-labeled.

- **TuELiP dataset:** The exact simulation method of TuELiP is described in its Supple-

simulation parameters	TuEliP	AncL2	p-value
k10,n5	0.064	0.064	0.540
k10,n10	0.058	0.055	0.057
k10,n20	0.056	0.057	0.320
k20,n5	0.048	0.049	0.770
k20,n10	0.05	0.05	0.158
k20,n20	0.046	0.046	0.692
k30,n5	0.049	0.049	0.215
k30,n10	0.042	0.042	0.759
k30,n20	0.044	0.043	0.124

Table 2: Comparison between mean CASet distance of 100 instances for each simulation settings between TuELiP and its single-label counterpart AncL2 algorithms on ConTreeDP original simulated data. The p-value is obtained from a paired t-test. There is no significant difference in the mean of distances with a p-value threshold of 0.05.

mentary Materials in detail. We have implemented this algorithm and simulated 900 samples of its instances for $n = 10, 20, 30$ and $k = 5, 10, 20$. The results reported for the TuELiP study are reproducible where it outperforms ConTreeDP and GraPhyC on this generated dataset. As it is depicted in Figure 5 of the TuELiP paper (Fig. 1), the GraPhyC method also has a better performance compared to ConTreeDP on this dataset and its average CASet distance is near zero in many instances.

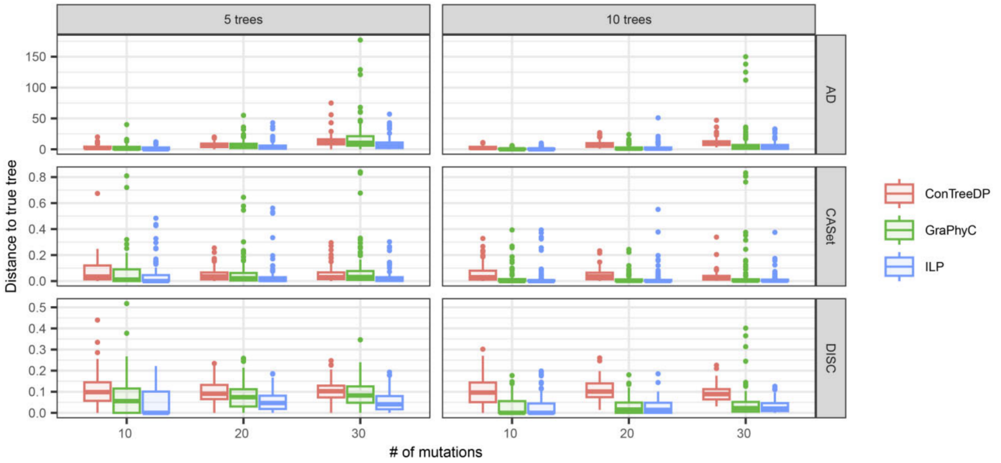

Figure 5. Results showing how well ConTreeDP, GraPhyC, and TuELiP uncover the true tumor trees for our simulated data. We measure the distances between the consensus trees and true trees using AD, CASet, and DISC.

Figure 1: Comparing TuELiP to ConTreeDP and GraPhyC from the [GSO23].

Based on these facts, we compared the performance of AncL2 to TuELiP on this dataset and here are our results:

– The average CASet distance between the outputs of these two algorithms is quite small (the minimum is 0.016, the mean is 0.027, and the max is 0.042).

– For the sake of comparison we also computed the average distance between GraPhyC's output and TuELiP's output (the minimum is 0.089, the mean is 0.109, and the max is 0.156), showing that the average distance between GraPhyC and TuELiP is more than four times greater than the average distance between AncL2 and TuELiP (Table 3).

– TuELiP finds the true ground truth tree in nearly 50% of the cases (Fig. 2) which is actually a multi-labeled tree.

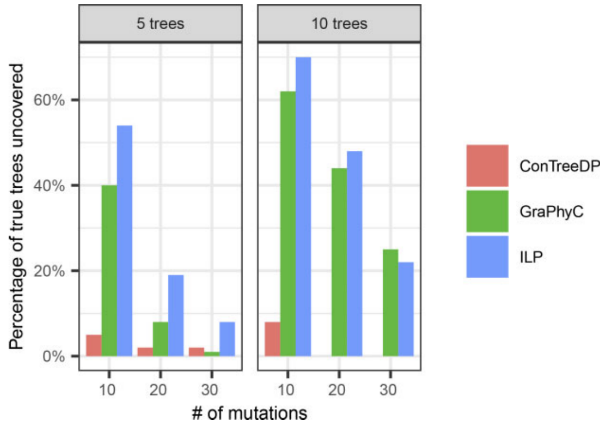

Figure 4. The results of counting the proportion of trials in which each method returned the exact true tree.

Figure 2: Number of found ground truth trees [GSO23].

Hence, one may conclude that AncL2 outperforms GraPhyC and ConTreeDP on this dataset, while it is weaker than TuELiP mainly when the ground truth trees are actually multi-labeled which is essentially an inevitable case.

simulation parameters	Average CASet distance between TuELiP & ground truth	Average CASet distance between AncL2 & ground truth	Average CASet distance between TuELiP & AncL2	Average CASet distance between TuELiP & GraPhyC
n10,k5	0.202	0.226	0.038	0.123
n10,k10	0.122	0.153	0.039	0.156
n10,k20	0.09	0.121	0.042	0.151
n20,k5	0.101	0.114	0.021	0.089
n20,k10	0.055	0.074	0.024	0.103
n20,k20	0.043	0.068	0.029	0.112
n30,k5	0.076	0.089	0.016	0.078
n30,k10	0.046	0.062	0.018	0.095
n30,k20	0.029	0.046	0.019	0.070

Table 3: Comparison between average CASet distance for 100 instances in 9 simulation scenarios between outputs of different methods and the ground truth trees.

9 Supplementary images and tables

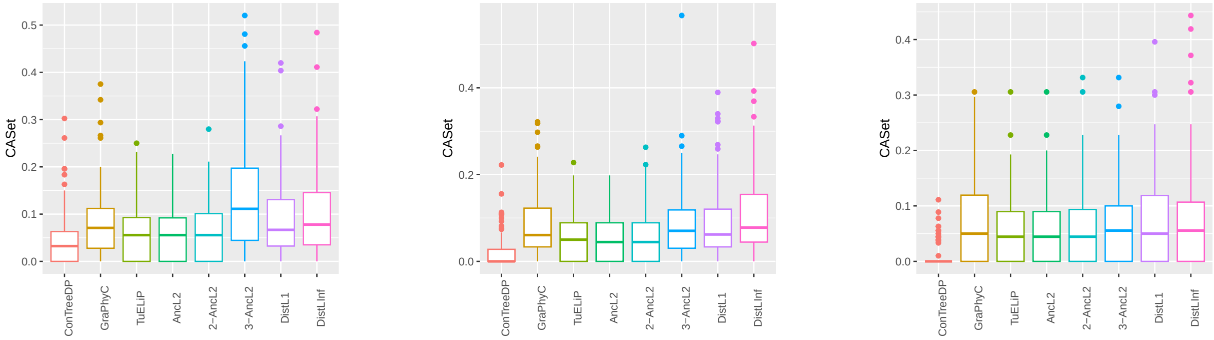

(a) $n = 10, k = 5$ (b) $n = 10, k = 10$ (c) $n = 10, k = 20$

Figure 3: Results of running different algorithms on ConTreeDP original simulated data. Clearly, ConTreeDP has the best performance on this dataset. It identifies the true structure of most of the trees.

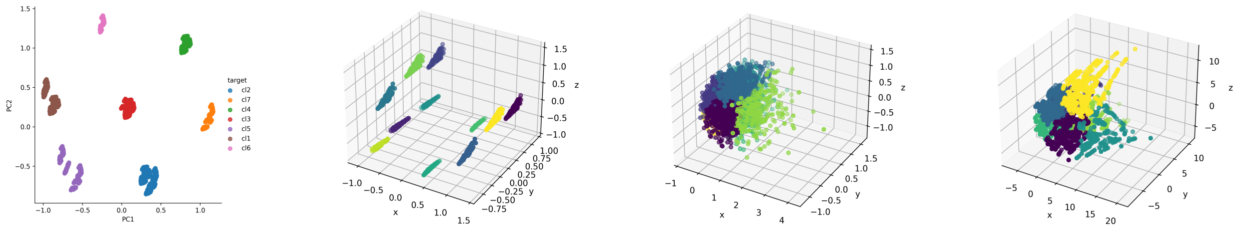

(a) 2D PCA for adjacency matrices of 4111 trees for patient P-0000041

(b) 3D PCA for adjacency matrices of 970 trees for patient P-0014476

(c) 3D PCA for ancestry matrices of 4111 trees for patient P-0000041

(d) 3D PCA for distance matrices of 4111 trees for patient P-0000041

Figure 4: PCA visualization for two patients using **adj**, **anc**, and **dist** mappings. Almost zero adjusted rand index shows low concordance between clusters of different mappings (table 4)
.

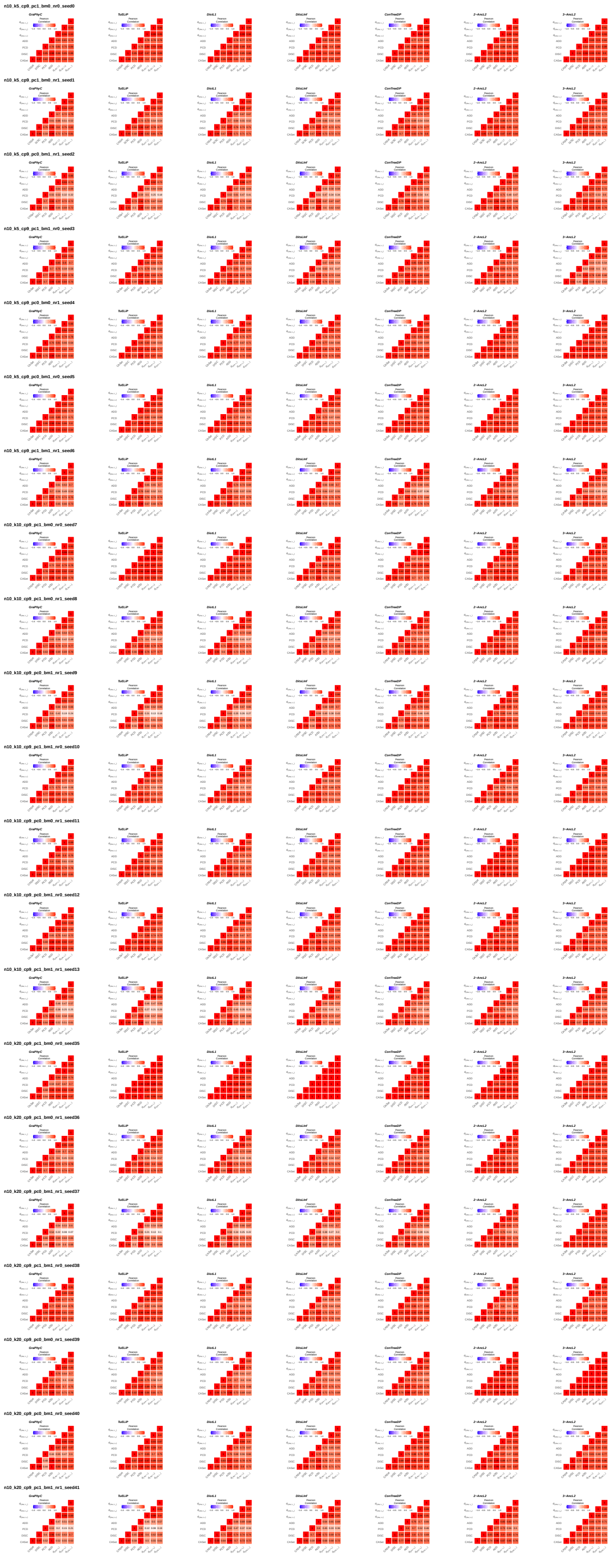

Figure 5: Pearson correlation between different distance measures for solutions of the same algorithm in various simulation settings. Each block shows Pearson correlation between different distance measures in computing the distance between solution trees and the ground truth trees for 100 samples of one specific mutation settings solved by a specific algorithm.

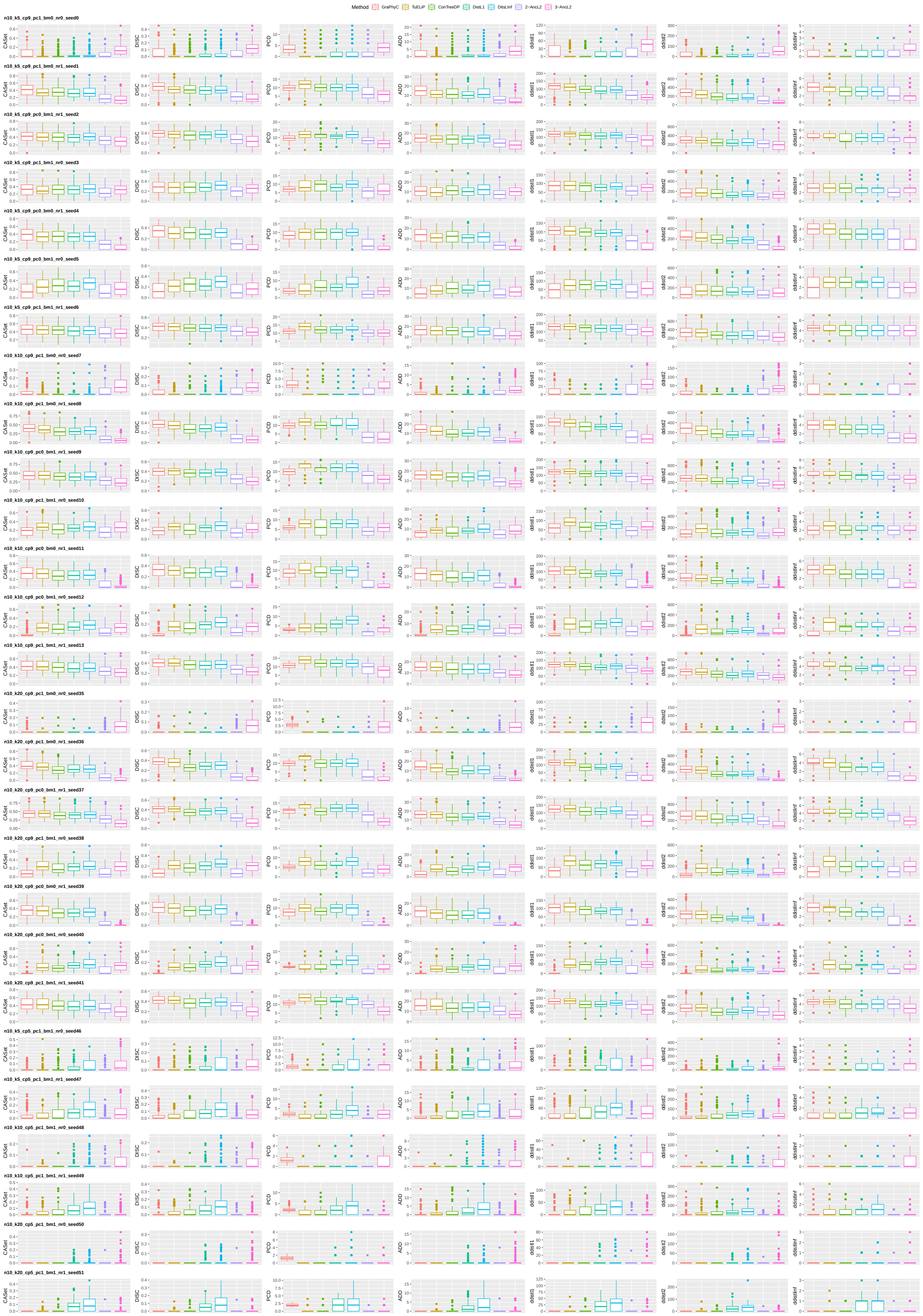

Figure 6: Distance to ground truth tree of outputs of different algorithms in different simulation settings on trees with 10 nodes. A single simulation setting with different distance measures are depicted in each row. Each block shows the box plot of distances between the solutions of different algorithms and the ground truth trees for 100 instances of a specific simulation settings computed by a specific distance measure.

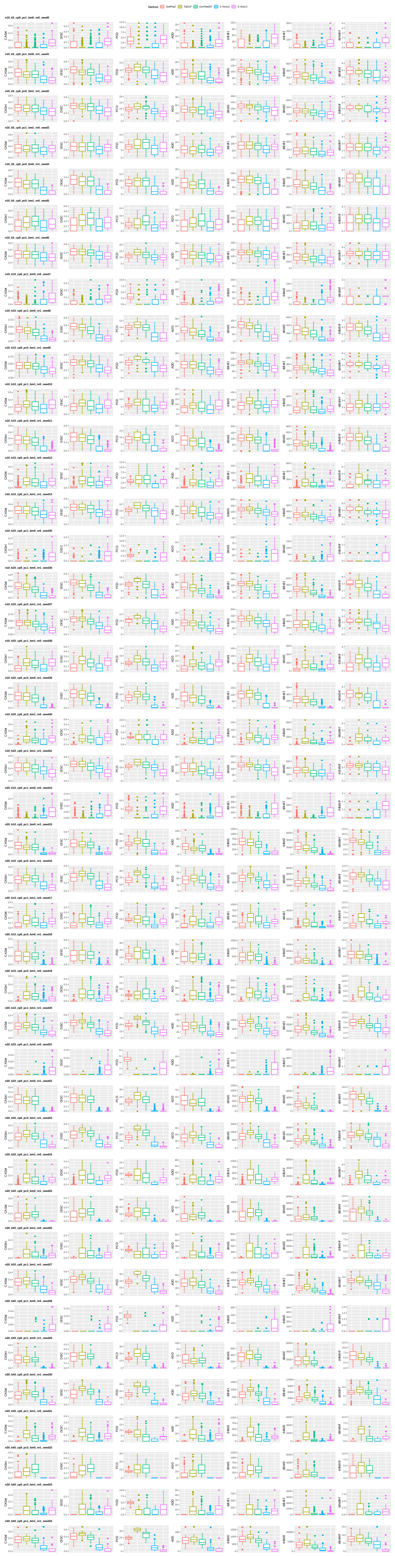

Figure 7: Distance to ground truth tree of outputs of ancestry based algorithms in different simulation settings on trees with 10 or 20 nodes. A single simulation setting with different distance measures are depicted in each row. Each block shows the box plot of distances between the solutions of different algorithms and the ground truth trees for 100 instances of a specific simulation settings computed by a specific distance measure.

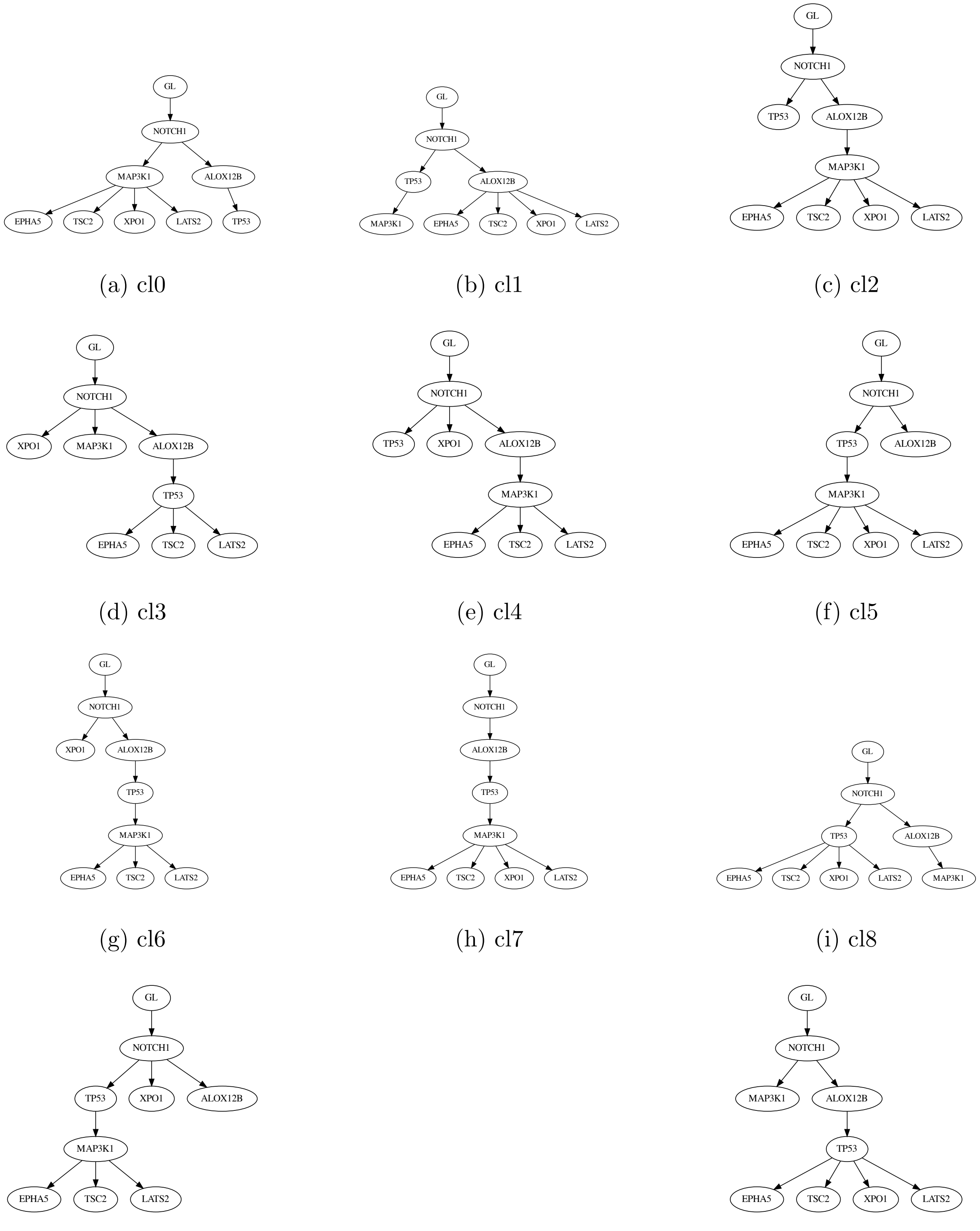

(a) cl0 (b) cl1 (c) cl2

(d) cl3 (e) cl4 (f) cl5

(g) cl6 (h) cl7 (i) cl8

(j) cl9 (k) cl10

Figure 8: Centroid trees for 11 clusters of adjacency matrices for patient P-0014476 using 2-AncL2 algorithm.

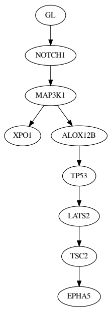

(a) cl0

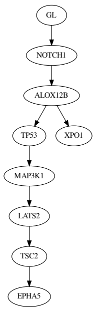

(b) cl1

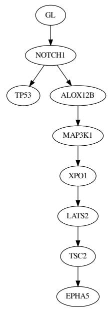

(c) cl2

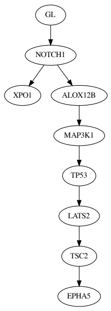

(d) cl3

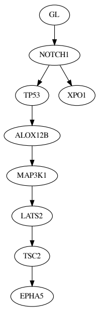

(e) cl4

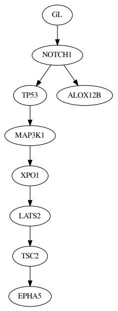

(f) cl5

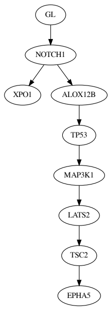

(g) cl6

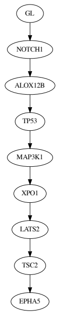

(h) cl7

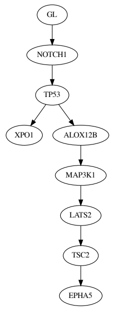

(i) cl8

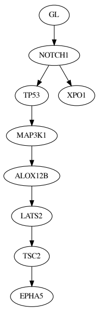

(j) cl9

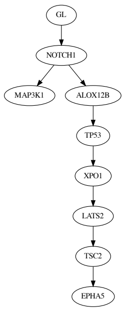

(k) cl10

Figure 9: Centroid trees for 11 clusters of adjacency matrices for patient P-0014476 using 3-AncL2 algorithm.

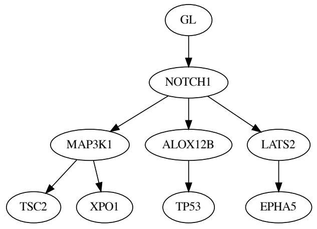

(a) cl0

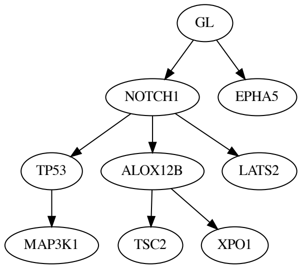

(b) cl1

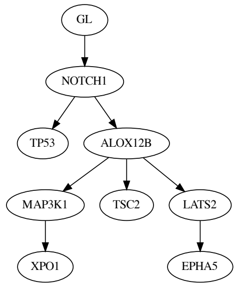

(c) cl2

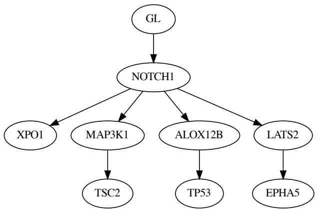

(d) cl3

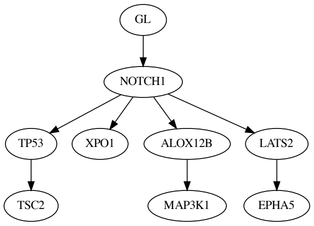

(e) cl4

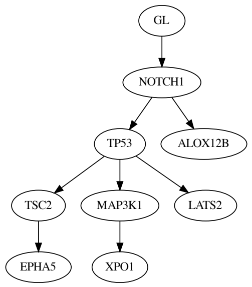

(f) cl5

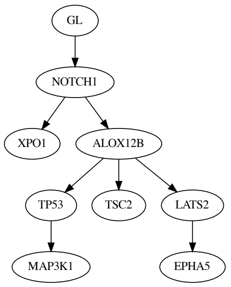

(g) cl6

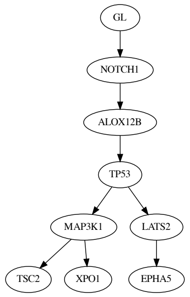

(h) cl7

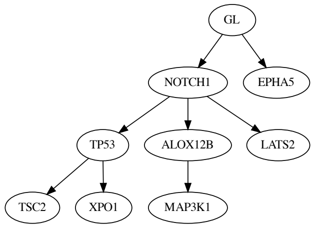

(i) cl8

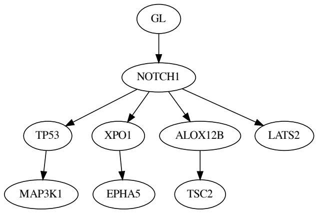

(j) cl9

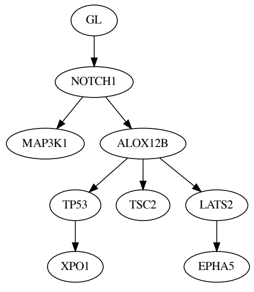

(k) cl10

Figure 10: Centroid trees for 11 clusters of adjacency matrices for patient P-0014476 using DistL1 algorithm.

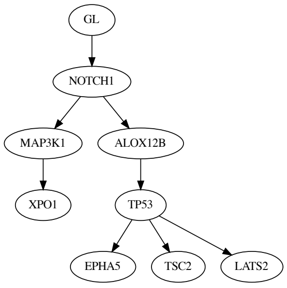

(a) cl0

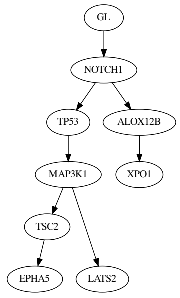

(b) cl1

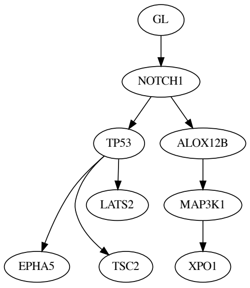

(c) cl2

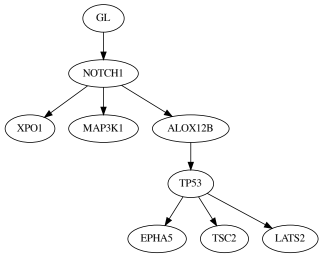

(d) cl3

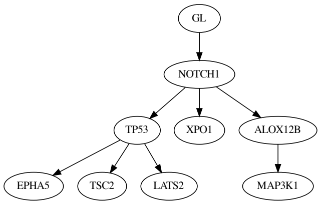

(e) cl4

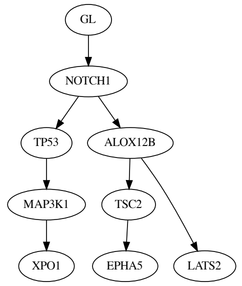

(f) cl5

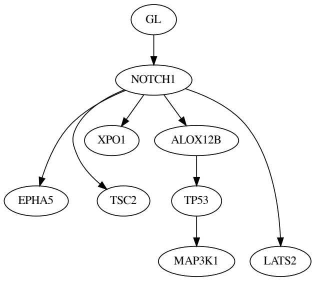

(g) cl6

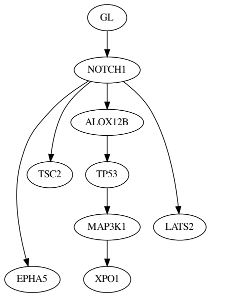

(h) cl7

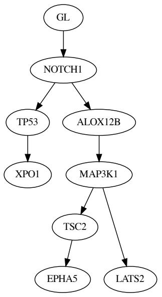

(i) cl8

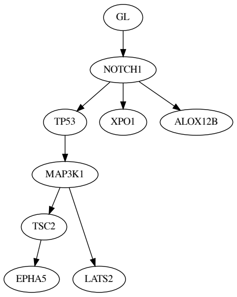

(j) cl9

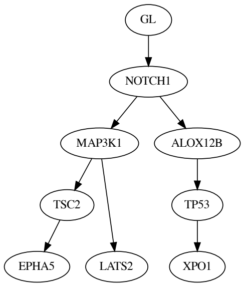

(k) cl10

Figure 11: Centroid trees for 11 clusters of adjacency matrices for patient P-0014476 using GraPhyC algorithm.

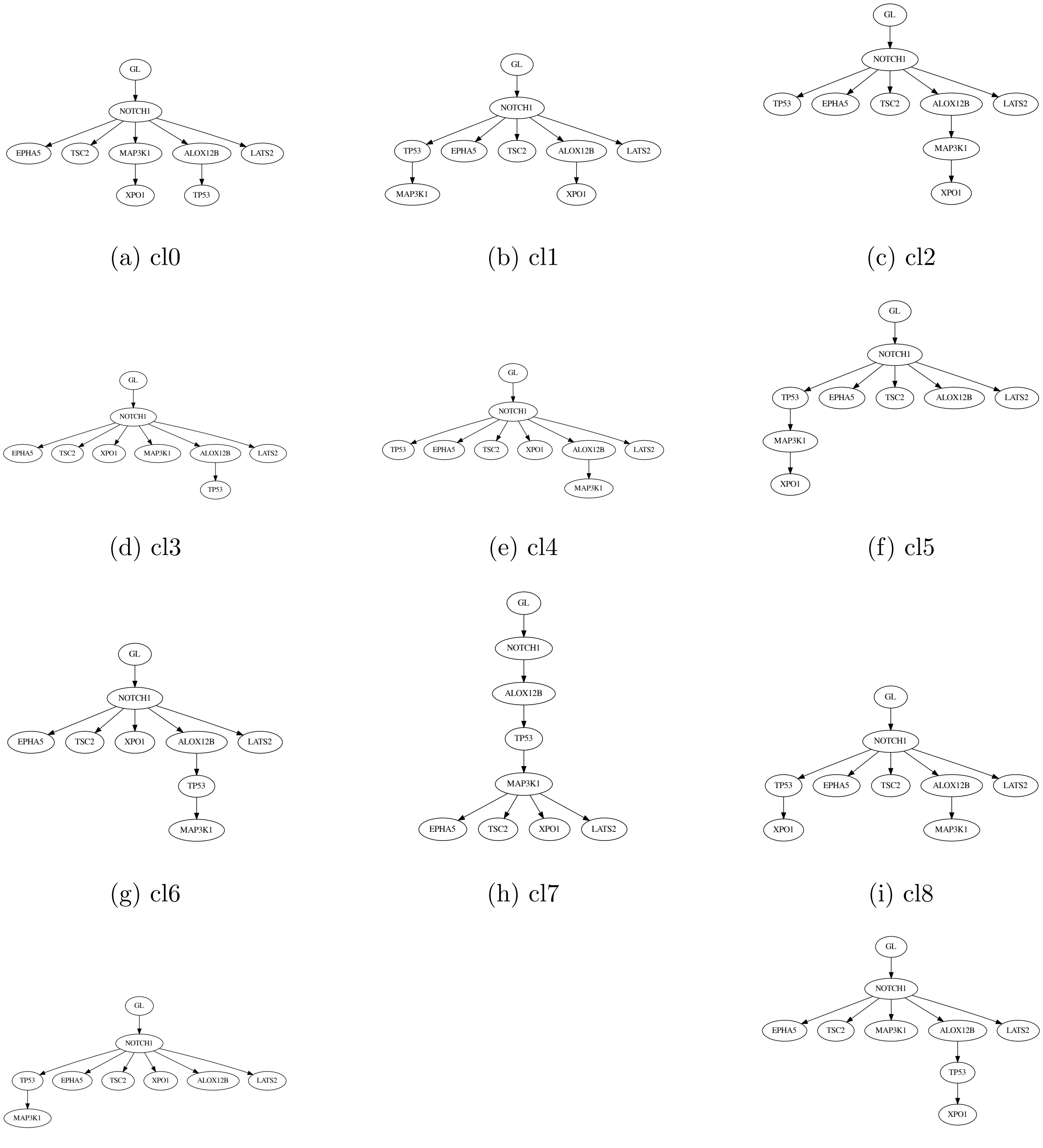

(a) cl0 (b) cl1 (c) cl2

(d) cl3 (e) cl4 (f) cl5

(g) cl6 (h) cl7 (i) cl8

(j) cl9 (k) cl10

Figure 12: Centroid trees for 11 clusters of adjacency matrices for patient P-0014476 using TuELiP algorithm.

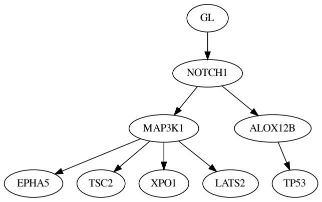

(a) cl0

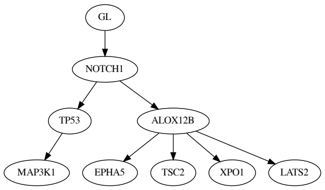

(b) cl1

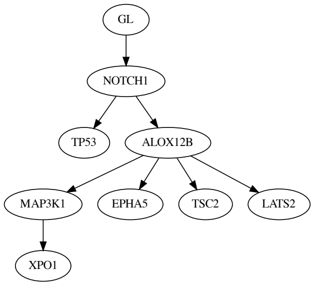

(c) cl2

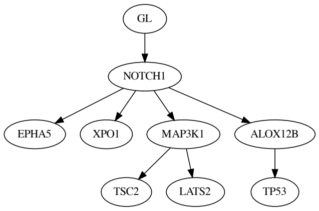

(d) cl3

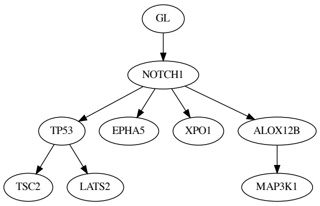

(e) cl4

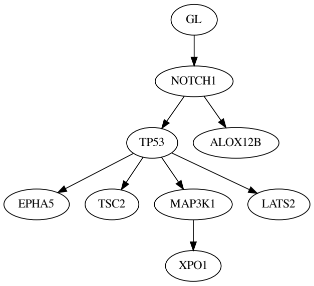

(f) cl5

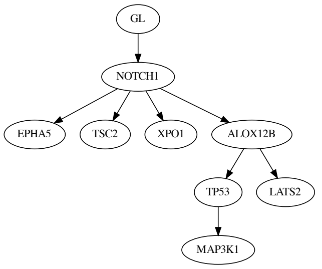

(g) cl6

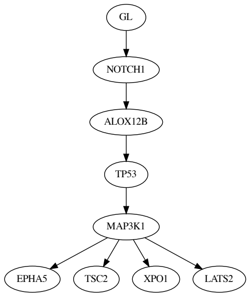

(h) cl7

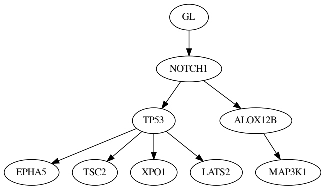

(i) cl8

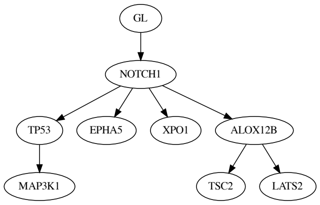

(j) cl9

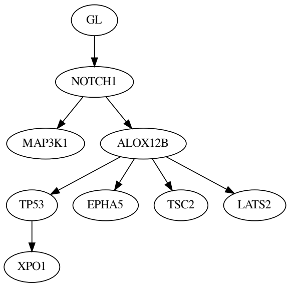

(k) cl10

Figure 13: Centroid trees for 11 clusters of adjacency matrices for patient P-0014476 using ConTreeDP algorithm.

	**adj** vs **anc**	**adj** vs **dist**	**anc** vs **dist**
Rand Index	0.76	0.75	0.77
Adjusted Rand Index	0.06	0.02	0.14

Table 4: Rand index and adjusted rand index for comparing cluster agreement between clusters of patient P-0000041's trees using adjacency, ancestry and distance mappings. Near zero adjusted rand index suggests that these clusters does not agree with each other more than any two random clustering of data with this sizes.

10 Acknowledgement

We would like to acknowledge that the chatbot functionality of Microsoft Bing was used in preparation of this manuscript. Bing provided assistance in enhancing the grammatical accuracy of the text.

References

[CL65] Yeong-Jin; Chu and Tseng-Hong Liu. "On the shortest arborescence of a directed graph". In: *Scientia Sinica* 14 (1965), pp. 1396–1400.

[Edm67] Jack Edmonds. "Optimum branchings". In: *Journal of Research of the National Bureau of Standards Section B Mathematics and Mathematical Physics* 71B.4 (Oct. 1967), p. 233.

[GJ79] Micheal R. ; Garey and David S. Johnson. *Computers and Intractability: A Guide to the Theory of NP-Completeness (Series of Books in the Mathematical Sciences)*. First Edit. W.H. Freeman, 1979.

[Gov+20] Kiya Govek et al. "GraPhyC: Using Consensus to Infer Tumor Evolution". In: *IEEE/ACM Trans. Comput. Biol. Bioinforma.* 19.1 (Jan. 2020), pp. 465–478.

[GSO23] Ziyun Guang, Matthew Smith-Erb, and Layla Oesper. "A weighted distance-based approach for deriving consensus tumor evolutionary trees". In: *Bioinformatics* 39.Supplement_1 (June 2023), pp. i204–i212.

simulation parameters	TuELiP	2-AncL2	p-value
n10,k5,cp9,pc1,bm0,nr0,seed0	0.03	0.054	0.000498802749637035
n10,k5,cp9,pc1,bm0,nr1,seed1	0.351	0.199	6.21553184301575e-23
n10,k5,cp9,pc0,bm1,nr1,seed2	0.415	0.319	3.832104141487e-10
n10,k5,cp9,pc1,bm1,nr0,seed3	0.316	0.243	5.36153827238589e-05
n10,k5,cp9,pc0,bm0,nr1,seed4	0.337	0.15	8.68349139426264e-26
n10,k5,cp9,pc0,bm1,nr0,seed5	0.263	0.174	1.66323151813889e-05
n10,k5,cp9,pc1,bm1,nr1,seed6	0.447	0.38	1.45154060241012e-09
n10,k10,cp9,pc1,bm0,nr0,seed7	0.025	0.049	4.54323949477723e-07
n10,k10,cp9,pc1,bm0,nr1,seed8	0.375	0.116	2.03934788282558e-36
n10,k10,cp9,pc0,bm1,nr1,seed9	0.454	0.3	2.93875270674626e-22
n10,k10,cp9,pc1,bm1,nr0,seed10	0.285	0.173	1.49718139031903e-09
n10,k10,cp9,pc0,bm0,nr1,seed11	0.344	0.083	2.60056492986411e-31
n10,k10,cp9,pc0,bm1,nr0,seed12	0.206	0.092	4.82467608534448e-11
n10,k10,cp9,pc1,bm1,nr1,seed13	0.437	0.31	8.04464188631585e-19
n10,k20,cp9,pc1,bm0,nr0,seed35	0.003	0.023	2.48168694659343e-05
n10,k20,cp9,pc1,bm0,nr1,seed36	0.391	0.1	1.17421515566041e-34
n10,k20,cp9,pc0,bm1,nr1,seed37	0.45	0.283	4.27865999480073e-24
n10,k20,cp9,pc1,bm1,nr0,seed38	0.251	0.101	6.05733736956944e-16
n10,k20,cp9,pc0,bm0,nr1,seed39	0.333	0.051	3.22702121930944e-38
n10,k20,cp9,pc0,bm1,nr0,seed40	0.172	0.072	1.81104059568273e-08
n10,k20,cp9,pc1,bm1,nr1,seed41	0.45	0.34	5.90376401932028e-20
n20,k10,cp9,pc1,bm0,nr0,seed14	0.014	0.019	2.20994686355396e-06
n20,k10,cp9,pc1,bm0,nr1,seed15	0.216	0.031	1.1443995325613e-34
n20,k10,cp9,pc0,bm1,nr1,seed16	0.331	0.151	8.89901865177825e-36
n20,k10,cp9,pc1,bm1,nr0,seed17	0.162	0.062	2.63149736339981e-15
n20,k10,cp9,pc0,bm0,nr1,seed18	0.185	0.014	5.53362066486002e-28
n20,k10,cp9,pc0,bm1,nr0,seed19	0.151	0.037	5.48740219740919e-14
n20,k10,cp9,pc1,bm1,nr1,seed20	0.337	0.158	5.39321658311198e-38
n20,k20,cp9,pc1,bm0,nr0,seed21	0	0.005	0.00178873285990913
n20,k20,cp9,pc1,bm0,nr1,seed22	0.217	0.006	7.68337320428402e-41
n20,k20,cp9,pc0,bm1,nr1,seed23	0.368	0.128	3.1541959482021e-44
n20,k20,cp9,pc1,bm1,nr0,seed24	0.166	0.036	5.02834009483885e-17
n20,k20,cp9,pc0,bm0,nr1,seed25	0.154	0.001	1.67446618205445e-25
n20,k20,cp9,pc0,bm1,nr0,seed26	0.114	0.026	1.55740400231217e-10
n20,k20,cp9,pc1,bm1,nr1,seed27	0.369	0.152	2.75946889991729e-37
n20,k40,cp9,pc1,bm0,nr0,seed28	0	0.002	0.0911862232401979
n20,k40,cp9,pc1,bm0,nr1,seed29	0.218	0.001	2.86312121595648e-39
n20,k40,cp9,pc0,bm1,nr1,seed30	0.378	0.106	6.84400767788821e-46
n20,k40,cp9,pc1,bm1,nr0,seed31	0.157	0.022	3.25648388301222e-21
n20,k40,cp9,pc0,bm0,nr1,seed32	0.137	0	1.60122792652538e-21
n20,k40,cp9,pc0,bm1,nr0,seed33	0.077	0.019	1.24997973448975e-08
n20,k40,cp9,pc1,bm1,nr1,seed34	0.381	0.129	6.07655772478256e-45

Table 5: Comparison between mean CASet distance of 100 instances for each simulation settings between TuELiP and 2-AncL2 algorithms. The p-value is obtained from a paired t-test. The threshold for p-values significance is 10^{-3}. The blue color shows smaller mean distance value (red is the opposite). The yellow color specifies insignificant p-values. The dominance of blue value in the 2-AncL2 column, is an evidence for its better performance.

simulation parameters	ConTreeDP	DistL1	p-value
n10,k5,cp9,pc1,bm0,nr0,seed0	0.037	0.032	0.220
n10,k5,cp9,pc1,bm0,nr1,seed1	0.353	0.337	0.084
n10,k5,cp9,pc0,bm1,nr1,seed2	0.399	0.392	0.447
n10,k5,cp9,pc1,bm1,nr0,seed3	0.322	0.327	0.617
n10,k5,cp9,pc0,bm0,nr1,seed4	0.345	0.325	0.017
n10,k5,cp9,pc0,bm1,nr0,seed5	0.288	0.279	0.380
n10,k5,cp9,pc1,bm1,nr1,seed6	0.436	0.424	0.143
n10,k10,cp9,pc1,bm0,nr0,seed7	0.014	0.008	0.179
n10,k10,cp9,pc1,bm0,nr1,seed8	0.318	0.326	0.288
n10,k10,cp9,pc0,bm1,nr1,seed9	0.415	0.408	0.386
n10,k10,cp9,pc1,bm1,nr0,seed10	0.231	0.262	0.009
n10,k10,cp9,pc0,bm0,nr1,seed11	0.305	0.308	0.708
n10,k10,cp9,pc0,bm1,nr0,seed12	0.169	0.215	1.4e-05
n10,k10,cp9,pc1,bm1,nr1,seed13	0.392	0.385	0.312
n10,k20,cp9,pc1,bm0,nr0,seed35	0.007	0.002	0.111
n10,k20,cp9,pc1,bm0,nr1,seed36	0.305	0.329	0.001
n10,k20,cp9,pc0,bm1,nr1,seed37	0.386	0.401	0.017
n10,k20,cp9,pc1,bm1,nr0,seed38	0.2	0.24	0.000
n10,k20,cp9,pc0,bm0,nr1,seed39	0.294	0.298	0.585
n10,k20,cp9,pc0,bm1,nr0,seed40	0.138	0.198	8.4e-09
n10,k20,cp9,pc1,bm1,nr1,seed41	0.39	0.393	0.663

Table 6: Comparison between mean CASet distance of 100 instances for each simulation settings between ConTreeDP and DistL1 algorithms. The p-value is obtained from a paired t-test and rounded to 3 decimal points. The threshold for p-values significance is 10^{-2}. The yellow color specifies insignificant p-values (green is the opposite). Dominance of yellow color suggests that these two algorithms have comparable performance.
